# Potential Contribution of Phenotypically Modulated Smooth Muscle Cells and Related Inflammation in the Development of Experimental Obstructive Pulmonary Vasculopathy in Rats

**DOI:** 10.1371/journal.pone.0118655

**Published:** 2015-02-25

**Authors:** Shoichiro Otsuki, Hirofumi Sawada, Noriko Yodoya, Tsutomu Shinohara, Taichi Kato, Hiroyuki Ohashi, Erquan Zhang, Kyoko Imanaka-Yoshida, Hideto Shimpo, Kazuo Maruyama, Yoshihiro Komada, Yoshihide Mitani

**Affiliations:** 1 Department of Pediatrics, Mie University Graduate School of Medicine, Tsu, Mie, Japan; 2 Department of Pediatrics, and Anesthesiology and Critical Care Medicine, Mie University Graduate School of Medicine, Tsu, Mie, Japan; 3 Department of Pediatrics and Neonatology, Nagoya City University Graduate School of Medical Sciences, Nagoya, Aichi, Japan; 4 Department of Pediatrics, Nagoya University Graduate School of Medicine, Nagoya, Aichi, Japan; 5 Department of Anesthesiology and Critical Care Medicine, Mie University Graduate School of Medicine, Tsu, Mie, Japan; 6 Department of Pathology, Mie University Graduate School of Medicine, Tsu, Mie, Japan; 7 Department of Thoracic and Cardiovascular Surgery, Mie University Graduate School of Medicine, Tsu, Mie, Japan; University of Nevada School of Medicine, UNITED STATES

## Abstract

We tested the hypothesis that phenotypically modulated smooth muscle cells (SMCs) and related inflammation are associated with the progression of experimental occlusive pulmonary vascular disease (PVD). Occlusive PVD was induced by combined exposure to a vascular endothelial growth factor receptor tyrosine kinase inhibitor Sugen 5416 and hypobaric hypoxia for 3 weeks in rats, which were then returned to ambient air. Hemodynamic, morphometric, and immunohistochemical studies, as well as gene expression analyses, were performed at 3, 5, 8, and 13 weeks after the initial treatment (n = 78). Experimental animals developed pulmonary hypertension and right ventricular hypertrophy, and exhibited a progressive increase in indices of PVD, including cellular intimal thickening and intimal fibrosis. Cellular intimal lesions comprised α smooth muscle actin (α SMA)+, SM1+, SM2+/-, vimentin+ immature SMCs that were covered by endothelial monolayers, while fibrous intimal lesions typically included α SMA+, SM1+, SM2+, vimentin+/- mature SMCs. Plexiform lesions comprised α SMA+, vimentin+, SM1-, SM2- myofibroblasts covered by endothelial monolayers. Immature SMC-rich intimal and plexiform lesions were proliferative and were infiltrated by macrophages, while fibrous intimal lesions were characterized by lower proliferative abilities and were infiltrated by few macrophages. Compared with controls, the number of perivascular macrophages was already higher at 3 weeks and progressively increased during the experimental period; gene expression of pulmonary hypertension-related inflammatory molecules, including IL6, MCP1, MMP9, cathepsin-S, and RANTES, was persistently or progressively up-regulated in lungs of experimental animals. We concluded that phenotypically modulated SMCs and related inflammation are potentially associated with the progression of experimental obstructive PVD.

## Introduction

Pulmonary arterial hypertension (PAH) is a progressive disease of the small pulmonary arteries characterized by obstructive intimal and plexiform lesions, and ultimately leads to right ventricular failure and premature death. Determining the cell type responsible for obstructive pulmonary vasculopathy is the basis for understanding the mechanisms involved and identifying the potential therapeutic target in the progressive vasculopathy in PAH. Previous pathological studies using human samples demonstrated that α-smooth muscle actin (αSMA)+, vimentin+ myofibroblasts or electron microscopy-based smooth muscle cell (SMC)-like cells, as well as inflammatory cells and apoptosis-resistant endothelial cells, may constitute such lesions in PAH, despite a controversy in such an issue. [1–4] It was recently shown that SMCs are a cell type that is not terminally differentiated and can retain remarkable plasticity. [5,6] Phenotypic modulation of SMCs in fact contributes to various physiological and pathological conditions, including development, tumor angiogenesis, and progression of vascular diseases such as atherosclerosis, aortic aneurysm, and restenosis after balloon injury. [5–10] Such immature SMCs may be relevant to the progression of pulmonary vasculopathy, because such modulation is associated with increased proliferation of SMCs and synthesis of extracellular matrix components, proteinases, cytokines, and angiogenic factors, which is typically accompanied by inflammatory cell infiltration. [5,6,11] In addition, the process of phenotypic modulation of SMCs is influenced by their interactions with endothelial cells, cytokines/growth factors (bone morphogenetic proteins, platelet-derived growth factor and transforming growth factor β), and CArG-serum response factor-myocardin-dependent transcriptional and epigenetic regulation in recent cell culture studies, which may be relevant to the development of PAH. [5,6,12,13] However, immature SMCs in these specific lesions have been poorly characterized, and how these SMCs, in concert with inflammatory cells, are associated with the progression of obliterative intimal and plexiform lesions in PAH is unknown.

To address these questions, it is important to use animal models with human PAH-like lesions, because of the limitations in obtaining tissue samples at various disease stages from patients with PAH. In addition, tissue samples, appropriately processed for immunohistochemical analyses with multiple SMC markers (ie, methanol-Carnoy’s fixed paraffin sections), may be required for the current phenotyping of SMC in vivo. [5,6,8–10,14,15] Recently, a new human PAH-like rat model accompanied by intimal and plexiform lesions, which mimic pulmonary vasculopathy in human PAH, was reported. [16] In this model, a single injection of a vascular endothelial growth factor (VEGF) receptor blocker Sugen 5416 in combination with chronic hypoxia for 3 weeks induced ‘progressive’ occlusive pulmonary vasculopathy with plexiform lesions, in contrast with ‘non-progressive’ pulmonary vasculopathy in rats exposed to chronic hypoxia alone. [17,18] Although apoptosis-resistant endothelial cells are believed to play a predominant role in the development of such obstructive pulmonary vasculopathy, [19,20] information regarding immature SMCs and inflammatory cells in these specific lesions, as well as related inflammatory gene expression in the lungs, is limited. This may preclude the opportunity to investigate the role of these cellular components in this model.

We therefore tested the hypothesis that immature SMCs, in concert with inflammatory cells, are temporally and topographically associated with the progression of occlusive and proliferative pulmonary vasculopathy in the Sugen/hypoxia model. Furthermore, we tested the hypothesis that expression of PAH-related inflammatory genes is distinctively up-regulated and differentially expressed in lungs in this progressive model, compared with in the non-progressive model induced by the exposure to chronic hypoxia alone.

## Methods

### Ethics Statement

Animal care, the experimental procedures, protocols for animal experiments were approved by the Animal Research Ethics Committee, Mie University School of Medicine (No. 24–9). All animal experiments were performed in accordance with the Guide for the Care and Use of Laboratory Animals published by the U.S. National Institute of Health (NIH Publication). Animals exposed to hypobaric hypoxia were subjected to twice a week cage cleaning and daily replenishment of food and water ad libitum. Catheterization and surgery were performed under sodium pentobarbital anesthesia, and all efforts were made to minimize suffering in the animal studies.

### Study design

Seven-week-old male Sprague-Dawley rats (Japan SLC, Inc., Shizuoka, Japan) were used for experiments, kept under standard laboratory conditions and fed a laboratory diet and water ad libitum. To establish experimental PAH, rats were injected subcutaneously with VEGF receptor tyrosine kinase inhibitor Sugen 5416 (20 mg/kg) (Sigma, St. Louis, MO) and exposed to hypobaric hypoxia (10% O_2_) for 3 weeks. [16,19,20] Rats were subsequently returned to ambient air (21% O_2_) and maintained for up to 10 weeks (total of 13 weeks after injecting Sugen 5416). [16,19,20] Rats were evaluated at 3, 5, 8 and 13 weeks after initial treatment. Hypoxic group rats were injected with diluent and were exposed to hypoxia for 3 weeks. Some hypoxic rats were returned to ambient air for additional 2 weeks (total of 5 weeks). Healthy control rats similarly received diluent and were maintained in ambient air for 3 or 5 weeks.

### Hemodynamic measurements and tissue preparation

The rats were anesthetized with pentobarbital sodium (33 mg/kg intraperitoneal). Right ventricular systolic pressure (RVSP) and mean systemic arterial pressure were measured after inserting a catheter of silicone elastomer tubing (inside diameter: 0.31 mm, outside diameter: 0.64 mm) through the right external jugular vein into the right ventricle, and through right carotid artery into ascending aorta by a closed-chest technique, as described previously. [18,21–23] RVSP and mean systemic arterial pressure were recorded using a physiological transducer (Uniflow, Baxter International Inc., Deerfield, IL), an amplifier system (AP-620G, Nihon Kohden, Tokyo, Japan) and a monitor (polygraph system, Nihon Kohden).

After hemodynamic measurements, lung tissue was prepared for vascular morphometry as described previously. [18,21–23] Briefly, after a rat was mechanically ventilated under pentobarbital sodium anesthesia, the lung was perfused through a pulmonary artery cannula with phosphate buffered saline. Next, the isolated lung was distended and fixed by perfusion through a tracheal tube with 4% phosphate-buffered paraformaldehyde for 3 hours and embedded into frozen sections, or placed in methanol-Carnoy’s solution, containing 60% (vol/vol) absolute methanol, 30% (vol/vol) chloroform, and 10% (vol/vol) glacial acetic acid for 4 hours before embedding into paraffin. Paraformaldehyde-fixed frozen sections and methanol-Carnoy’s fixed paraffin sections, obtained from the midsection of the left lung, were used for morphometric analysis, immunohistochemistry and immunofluorescence. For histological analysis, 5-μm sections were prepared. Right ventricle was dissected from the left ventricle plus the septum and weighed separately. The weight ratio of the right ventricle to the left ventricle + septum (RV/LV+S) was calculated.

### Histological and morphometric analysis

Cellular intimal thickening was identified by the characteristic proliferating intimal cellular masses that stain brown, and intimal fibrosis was identified by concentric or eccentric intimal masses of less cellular fibrous tissue that stain bright red in elastic van Gieson staining. [24] The lesions at the earliest stages of histological lesions observed, with respect to intimal formation were described as ‘sprouting’ intimal lesions. Plexiform lesion was identified by a sac-like complex lesion, including a cluster of cellular mass, which projected out of the parent artery into the lung parenchyma and was covered by fragments of single fragile elastic lamina or thin layer of muscle between 2 ill-defined elastic laminae. [24] The lesions at the earliest stages of histological lesions observed, with respect to the sac-like complex formation were described as ‘sprouting’ plexiform lesions. A vessel in which the lumen was partially (> 50%) or fully obstructed was defined as an occlusive lesion. [25] Quantitative analysis was performed to determine the proportion of such occlusive vessels among all the small pulmonary arteries (outer diameter: 15–50 μm) per lung section.[25] Quantitative analysis was performed to determine the relative proportion of vessels accompanied by cellular intimal thickening or intimal fibrosis among all the pulmonary arteries (outer diameter; 15–200 μm) per lung section, as the diameter of some vessels with intimal lesions, especially intimal fibrosis, is > 50 μm. [16] The external diameter of small pulmonary arteries in the lung section was measured along the shortest curvature. [18,21–23]

### Immunohistochemistry and histochemistry

Methanol-Carnoy’s fixed paraffin sections were deparaffinized and rehydrated. Epitope retrieval was performed by boiling the sections in citrate buffer (pH 6.0). Sections were reacted with 0.3% hydrogen peroxide to block endogenous peroxidase and blocked with 1% bovine serum albumin in phosphate buffered saline. [8–10,15] Sections were then incubated with primary antibodies overnight at 4°C. After streptavidin-biotin amplification (LSAB2 kit, DAKO, Carpinteria, CA), the slides were incubated with 3, 3’-diaminobenzidine and counterstained with hematoxylin. A negative control was performed using isotype-matched mouse IgG or rabbit immunoglobulin (DAKO), instead of the primary antibody. The localization and intensity of immunoreactivity were determined by an investigator, who was blinded to the experimental and control groups. Primary antibodies were as follows: αSMA (mouse monoclonal 1A4, Sigma), SM1 (mouse monoclonal 1C10, Yamasa, Tokyo, Japan), SM2 (mouse monoclonal 1G12, Yamasa), HHF35 (mouse monoclonal, Enzo Life Sciences, Plymouth Meeting, PA), CGA7 (mouse monoclonal, Enzo Life Sciences), von Willebrand factor (VWF) (rabbit polyclonal, Millipore, Billerica, MA), CD68 (mouse monoclonal ED1, Millipore), CD3 (rabbit monoclonal SP7, abcam), proliferating cell nuclear antigen (PCNA) (mouse monoclonal PC10, DAKO), tenascin C (gift from Prof. K Imanaka-Yoshida), vimentin (mouse monoclonal V9, DAKO), matrix metalloproteinase 9 (MMP9) (mouse monoclonal GE-213, Lab Vision), interleukin 6 (IL6) (rabbit polyclonal, Abcam, Camridge, UL), and monocyte chemotactic protein 1 (MCP1) (rabbit polyclonal, Abcam). Methanol-Carnoy’s fixed paraffin sections were prepared for immunohistochemical studies using SM1, SM2, HHF35, and CGA7 since this has been optimized for such preparations in a series of previous studies. [8–10,14,15,26] Paraformaldehyde-fixed frozen sections were used for immunohistochemical analysis for cathepsin S (rabbit polyclonal, Novus, Littleton, CO) and regulated on activation and normal T cell expressed and secreted (RANTES) (rabbit polyclonal, Novus Biologicals). Toluidine Blue staining for mast cells was performed by incubating deparaffinized and rehydrated sections for 5 minutes in a working solution of stock Toluidine Blue (0.05% Toluidine Blue Solution pH4.1, Wako), followed by dehydration and mounting with mounting media.

### Assessment of phenotypically modulated smooth muscle cells

SMCs in vascular lesions were immunophenotyped using two anti-SMC myosin heavy chain markers (SM1 and SM2) and three anti-actin markers (αSMA, HHF35, and CGA7). Cells that stained positive (+) for SM1, SM2, αSMA, HHF35, and CGA7 were considered to be mature SMCs; in contrast, those staining positive for SM1, HHF35, and αSMA but weakly positive (+/-) or negative (-) for SM2 or CGA7 were considered to be phenotypically modulated immature SMCs (S1 Table). [8–10,14,15] To quantitatively analyze the proportion of mature or immature SMC-dominant lesions in EVG-defined occlusive lesions with cellular intimal thickening or intimal fibrosis, mature or immature SMC-dominant lesion was defined as the lesion in which mature or immature SMC is the predominant (>50%) phenotype of SMC on the basis of SM1 and SM2 staining.

### Quantitative analysis of perivascular and intimal infiltration of macrophages, T cells, and mast cells

Quantitative analysis of perivascular inflammatory cells was performed by counting CD68-positive macrophages, CD3-positive T cells, and mast cells, surrounding all pulmonary arteries (external diameter; 15–200 μm), per vessel in an entire lung section. [23] Quantitative analysis of macrophage infiltration of intima was performed by determining the percentage of intimal lesions including at least one CD68-positive macrophage among all the intimal lesions in an entire lung section.

### Immunofluorescent staining

Five-μm thick frozen or paraffin sections were incubated with primary antibodies that recognize anti-VWF, anti-PCNA, anti-CD68, anti-MMP9 and anti-MCP1 overnight at 4°C, followed by incubation with Alexa 488 (goat anti-mouse)-conjugated secondary antibody (Molecular Probes, Eugene, OR). Next, sections were stained with mouse anti-Cy3-conjugated αSMA (Sigma) and TO-PRO-3 iodide (Molecular Probes, Eugene, OR) to visualize nuclei. Vessels were assessed using fluorescence and confocal microscopy (FV1000, Olympus, Tokyo, Japan). [23]

### Quantitative real time-polymerase chain reaction (PCR)

Total RNA was extracted and purified from whole lung tissue using RNAeasy Mini kits (QIAGEN, Valencia, CA). The quantity and quality of RNA was determined by using a spectrophotometer: 2 μg of RNA was used as template for reverse transcription PCR with the cDNA Synthesis Kit (Invitrogen, Carlsbad, CA). Quantitative real-time PCR was performed on a StepOnePlus Real Time PCR System with TaqMan^R^ Gene Expression Assays on Demand probes. PCR primers were as follows: interleukin 1β (IL1β) Rn00580432_m1, IL6 Rn01410330_m1, tumor necrosis factor α (TNFα) Rn01525859_g1, MCP1 Rn00580555_m1, RANTES Rn00579590_m1, vascular endothelial growth factor A (VEGF A) Mm01281449_m1, Hypoxanthine Phosphoribosyltransferase 1 (HPRT1) Rn01527840_m1, matrix metalloproteinase 2 (MMP2) Rn01538170_m1, MMP9 Rn00579162_m1, cathepsin S Rn00569036_m1, tissue inhibitor of metalloproteinase 1 (TIMP1) Rn01430873_g1, tissue inhibitor of metalloproteinase 2 (TIMP2) Rn00573232_m1 (Applied Biosystems, Foster City, CA). Relative standard curve values were determined with StepOne software, and values were normalized against HPRT1. Data are expressed as fold-change compared with the control group.

### Statistics

Graphical and statistical analyses were performed using GraphPad Prism 6 (GraphPad, San Diego, CA). Hemodynamic and morphological parameters, the number of macrophages, and mRNA levels were compared among ≥3 study groups or various time points with a one-way analysis of variance followed by Tukey-Kramer multiple comparison test. The correlation between the percentage of occlusive lesions and RVSP or the number of perivascular macrophages was analyzed with Pearson product-moment correlation coefficients. The proportion of immature SMC-dominant lesions in occlusive lesions with cellular intimal thickening and in those with intimal fibrosis was compared by chi-square analysis. Values are shown as mean ± standard deviation (SD). A value of *p*<0.05 was accepted as statistically significant.

## Results

**Fig 1:**
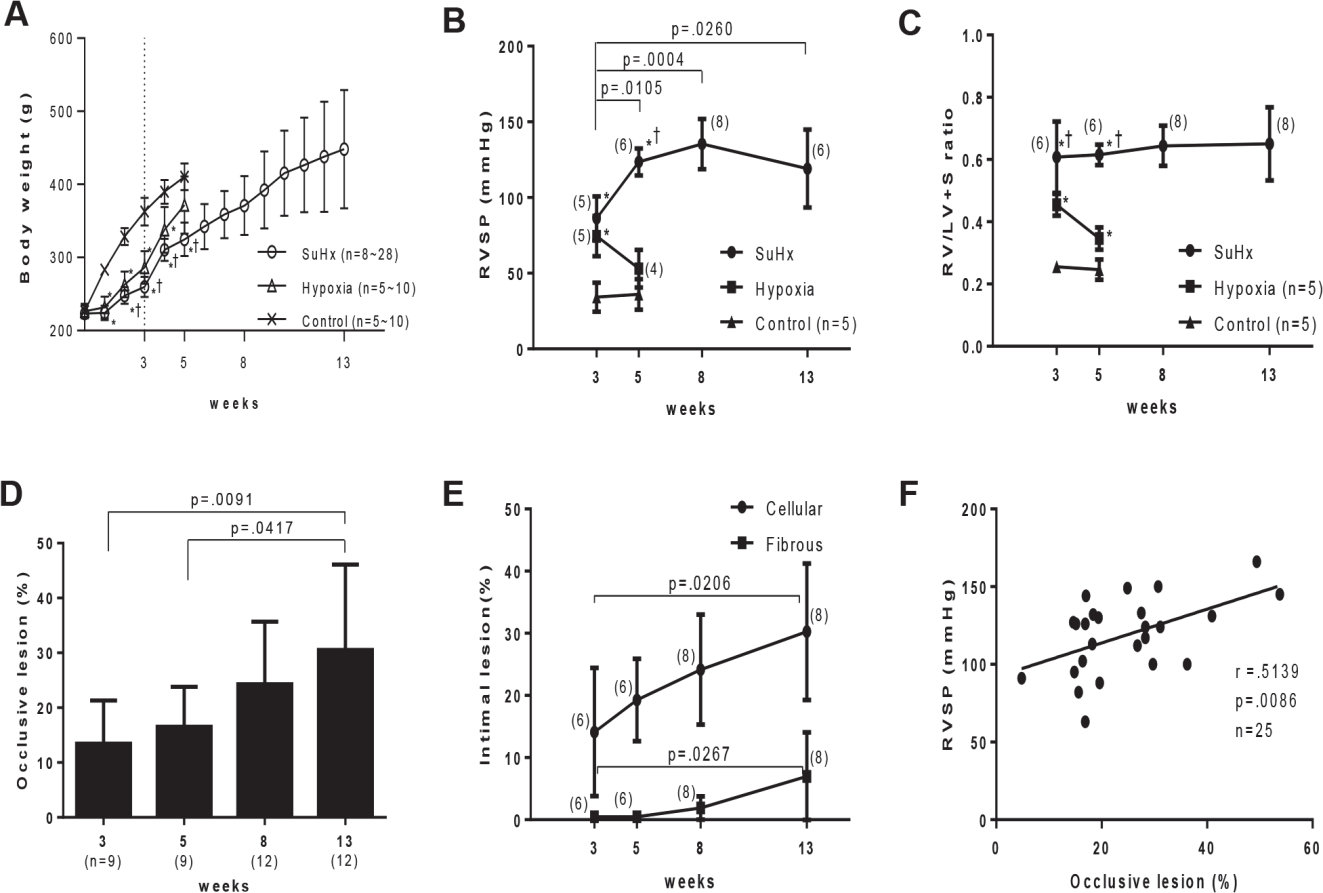
Progression of pulmonary hypertension and an occlusive pulmonary vasculopathy in Sugen/hypoxia rats. Effects of Sugen/hypoxia (SuHx) or hypoxia on body weight (A), right ventricular systolic pressure (RVSP) (B), the weight ratio of the right ventricle to the left ventricle + septum (RV/LV+S) (C), percentage of vessels accompanied by occlusive lesions among the small pulmonary arteries per lung section (D), and percentage of vessels accompanied by cellular intimal thickening or intimal fibrosis in all the small pulmonary arteries per lung section (E) during the experimental period. Body weight, RVSP, and R/VL+S ratio among 3 study groups at each time point (A, B, C) or these hemodynamic and morphological parameters at various time points (B, C, D, E) were compared with a one-way analysis of variance followed by Tukey-Kramer multiple comparison test. *P*<.05 vs. control; †*P*<.05 vs. hypoxia. F: Correlation between the percentage of occlusive lesions and RVSP (Pearson product-moment correlation coefficients). Values are mean ± SD.

All the experimental and control rats survived during the experimental period. Body weight in Sugen/hypoxia rats and in hypoxic rats was significantly lower than in control rats at during 1–5 weeks; body weight in SuHx rats was significantly lower than in hypoxic rats during 2–5 weeks (Fig. 1A, S2 Table). Mean systemic arterial pressure in Sugen/hypoxia and hypoxic rats was comparable to that in controls at the respective time point (data not shown).

### Progression of pulmonary hypertension and an occlusive pulmonary vasculopathy in Sugen/hypoxia rats

Three weeks after initial treatment, RVSP was significantly higher in Sugen/hypoxia (86 ± 14 mmHg, p < .0001) and in hypoxic rats (74 ± 13 mmHg, p = .0008), than in controls (34 ± 9 mmHg) (Fig. 1B). RVSP in Sugen/hypoxia rats increased progressively from 3 to 8 weeks (135 ± 16 mmHg vs. 3 weeks, p = .0004), while RVSP in hypoxic rats returned to control levels at 5 weeks (53 ± 12 mmHg vs. hypoxic rats at 3 weeks, p = .0420; vs. control rats at 5 weeks, p = .0709). At 3 weeks, Sugen/hypoxia rats exhibited more severe right ventricular hypertrophy (RV/LV+S, 0.60 ± 0.11) than hypoxic (0.45 ± 0.03, p = .0119) and control rats (0.25 ± 0.01, p < .0001) (Fig. 1C). The level of right ventricular hypertrophy in Sugen/hypoxia rats remained constant until 13 weeks (0.65 ± 0.11), while the level of right ventricular hypertrophy in hypoxic rats improved at 5 weeks (0.34 ± 0.03 vs. hypoxic rats at 3 weeks, p = .0012; vs. controls (0.24 ± 0.03) at 5 weeks, p = .0011).

In histological studies, the percentage of occlusive lesions in Sugen/hypoxia rats was already significantly higher at 3 weeks (13.4 ± 7.8% vs. 0.0 ± 0.0% in controls, p = .0015) (data not shown), and progressively increased until 13 weeks (30.5 ± 15.5%, p = .0091 vs. 3 weeks) (Fig. 1D). The percentage of vessels with the cellular intimal thickening (14.1 ± 10.3% vs. 0.0 ± 0.0% in controls, p = .0090) and with intimal fibrosis (0.4 ± 0.6% vs. 0.0 ± 0.0% in controls, p = .1438) were higher at 3 weeks (data not shown) and significantly increased until 13 weeks (30.2 ± 10.9%, p = .0206 and 6.9 ± 7.0%, p = .0267, respectively) (Fig. 1E). The diameter of vessels with these intimal lesions was mostly ≤ 50 μm: in 97.3% of vessels with cellular intimal thickening and 92.2% of vessels with intimal fibrosis. The percentage of occlusive lesions was positively correlated with RVSP (p = .0086, r = .5139) (Fig. 1F).

### Topography of intimal thickening and plexiform lesions

**Fig 2:**
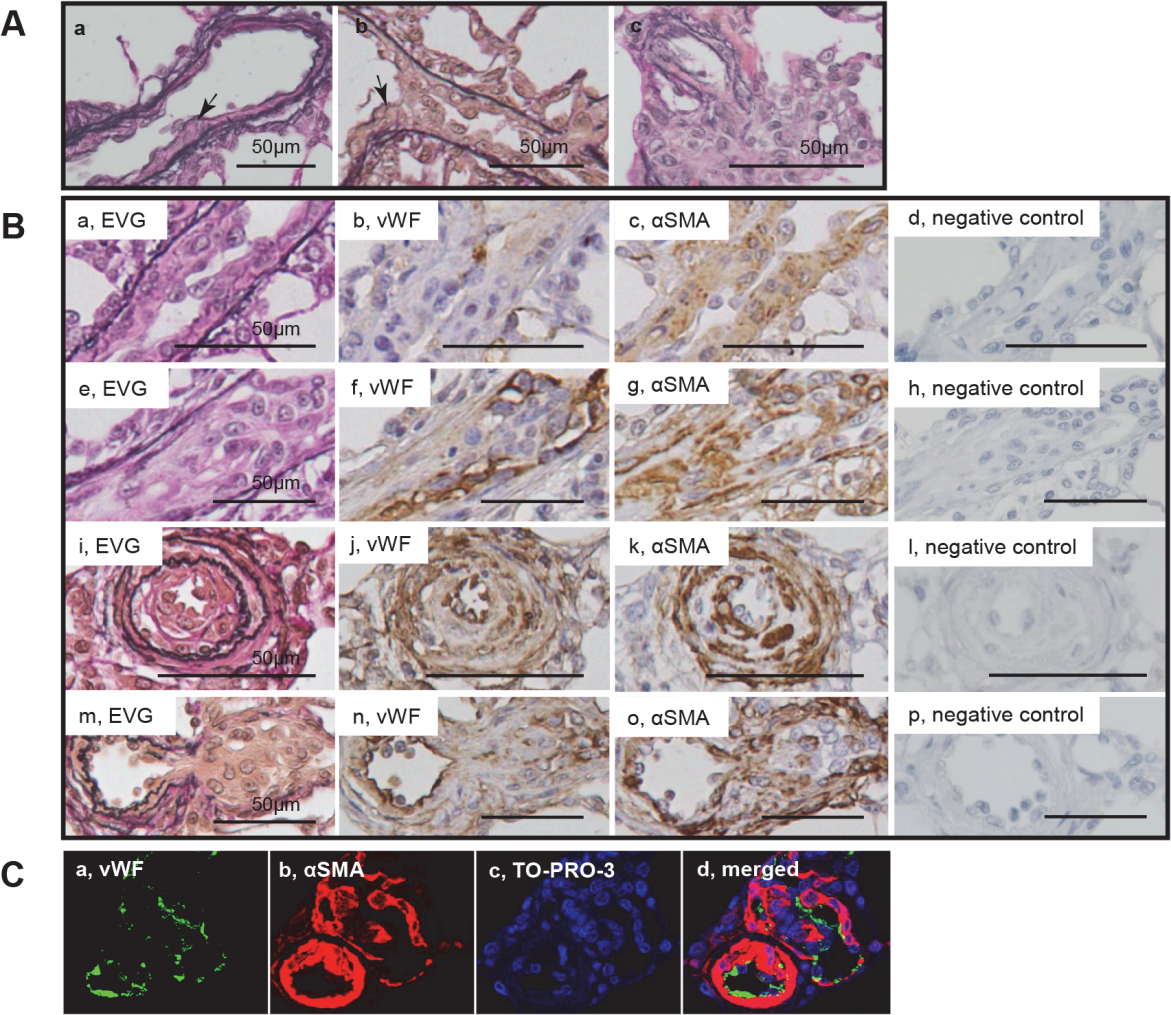
Cellular components in intimal and plexiform lesions. Photomicrographs (elastic van Gieson staining, EVG) of sprouting intimal lesion (Panels Aab) and sprouting plexiform lesion (Panel Ac) in rats 3 weeks after initial treatment. Serial sections of longitudinal views (Panels Ba-h) and cross sectional views (Panels Bi-l) of intimal lesions and serial sections of plexiform lesions (Panels Bm-p) in EVG, von Willebrand factor (VWF), α-smooth muscle actin staining (αSMA) and negative controls. Photomicrographs (confocal microscopy) of a section of the intimal and plexiform lesion (Panels C), by using antibodies for VWF and αSMA, were presented. TO-PRO-3, nuclear staining. Arrows indicate abnormal cells.

**Fig 3:**
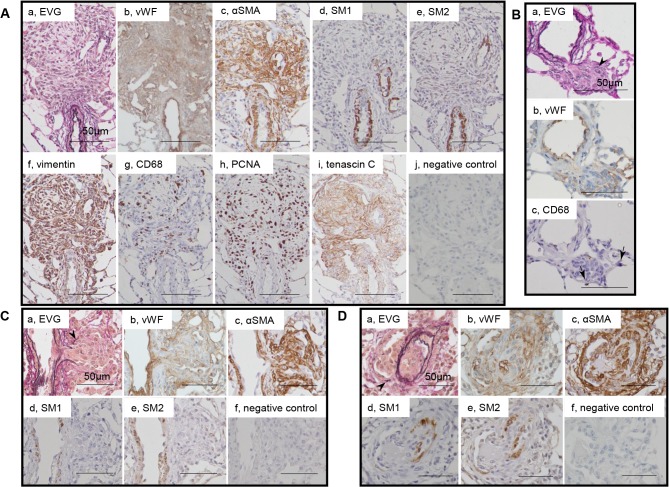
Myofibroblasts in plexiform lesions. Photomicrographs of serial sections of complex plexiform lesions in a rat 13 weeks after initial treatment (Panels A) and sprouting plexiform lesions (Panels B, C, D). Immunohistochemical findings using antibodies for various antibodies were presented. αSMA+, vimentin+ supporting cells, which underlies endothelial monolayers, were regarded as myofibroblasts and were negative for SM1 or SM2. PCNA indicates proliferating cell nuclear antigen. An arrow head indicates fragments of elastic laminae. Abbreviations are described in Fig. 2.

In the longitudinal and cross sections of intimal lesions, an intimal cell mass occluded the vessel lumen (Fig. 2Aab, Ba-l). VWF-positive endothelial cells formed a luminal monolayer, which covered the supporting ‘hyperchromatic and oval’ cells positive for αSMA in the intima-media complex (Fig. 2B, C). Sprouting intimal lesions, which were observed as early as 3 weeks, were associated with the fragmented internal and external elastic laminae. (Fig. 2Ab, Ba). The complex plexiform lesion, which was observed predominantly 13 weeks after initial treatment, comprised a plexus of aneurysmal and angiomatoid small vessels and supporting cellular and matrix compontents, was covered by remnants of elastic laminae. (Fig. 3A, S1A-B Fig.). The ‘sprouting’ plexiform lesion, which began to be observed even rarely as early as 3 weeks, was a focal projection originating from the parent vessel and frequently appeared to be located within an aneurismal dilatation of a side branch with fragmented elastic laminae (Fig. 2Ac, Bm, Fig. 3B, C, D). VWF-positive endothelial cells formed a monolayer on the luminal surface in the vascular channel-like structure, that was supported by αSMA+ supporting cells (Fig. 2Bm-p, C); in some complex plexiform lesions, endothelial monolayers covering vascular channel lumen were sparsely distributed and were separated by abundant αSMA+ supporting cell cluster and matrix deposition (Fig. 3A, S1C-D Fig.). αSMA+ dilatation lesions in plexiform lesions appeared to be contiguous with αSMA + media of the parent vessels, while αSMA+ supporting cells in plexiform lesions were continuous with αSMA+ supporting cells in the intima of some parent vessels (Fig. 3C, D).

### Phenotypically modulated smooth muscle cells in intimal lesions

**Fig 4:**
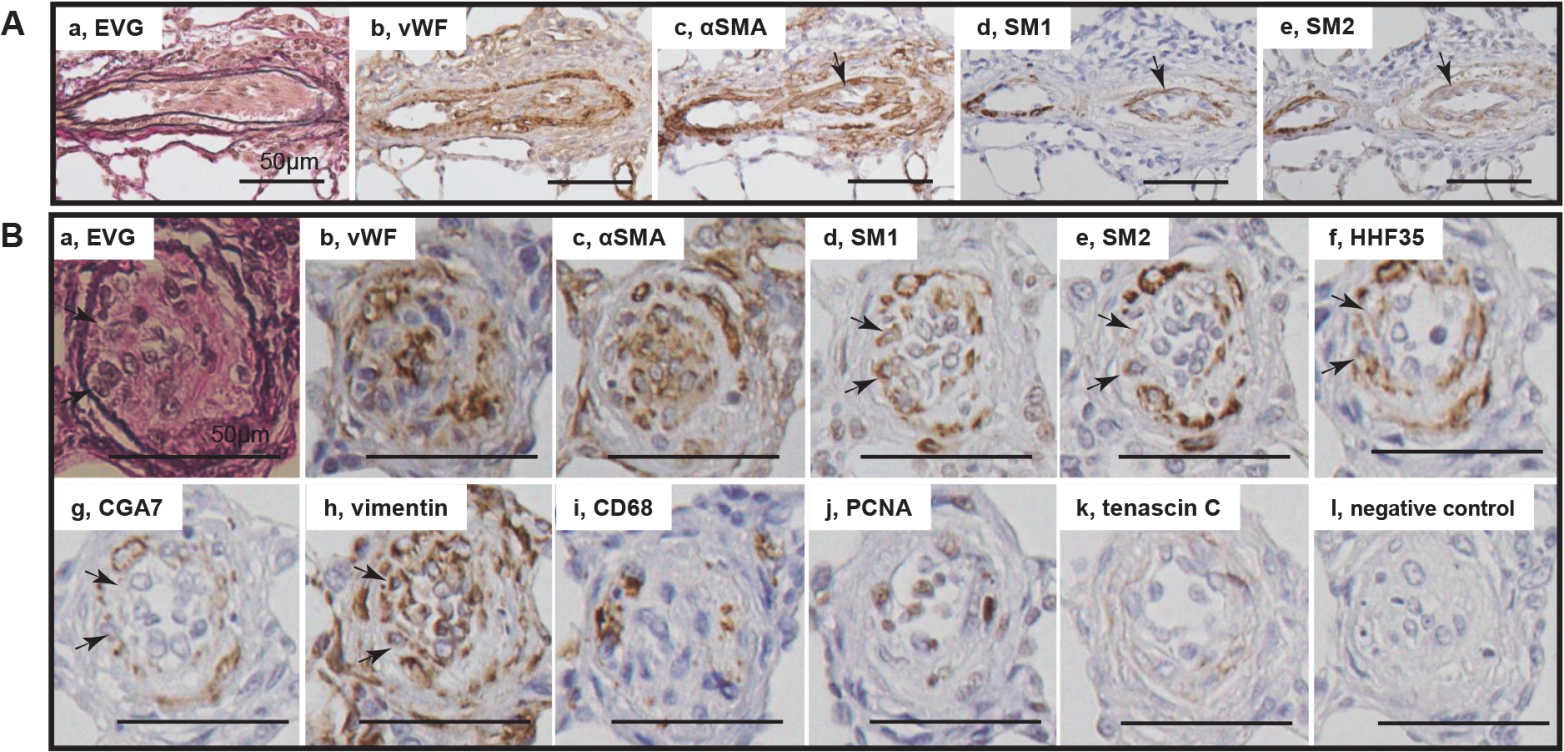
Immature smooth muscle cells in cellular intimal lesions. Photomicrographs of serial sections of cellular intimal lesions (Panels A, B). Cells staining positive for αSMA, SM1 and HHF35 but weakly positive or negative with SM2 and CGA7 were regarded as representing phenotypically modulated immature smooth muscle cells. Arrows indicate immature smooth muscle cells. Other abbreviations are described in Figs. 2 and 3.

**Fig 5:**
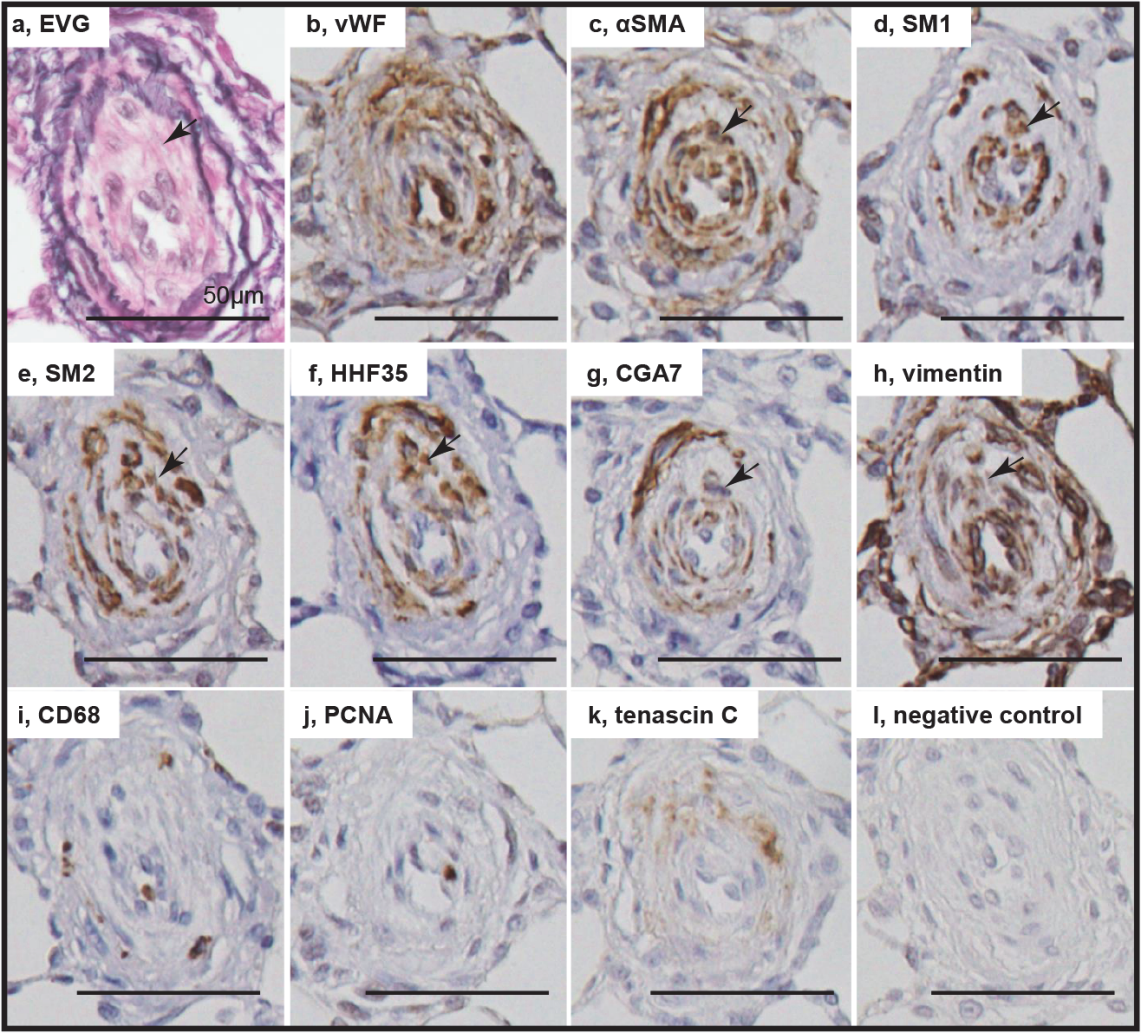
Mature smooth muscle cells in intimal fibrosis. Photomicrographs of serial sections of intimal fibrosis. Cells staining positive for αSMA, SM1, SM2, HHF35 and CGA7 were regarded as representing mature smooth muscle cells. Arrows indicate mature smooth muscle cells. Abbreviations are described in Figs. 2 and 3.

**Fig 6:**
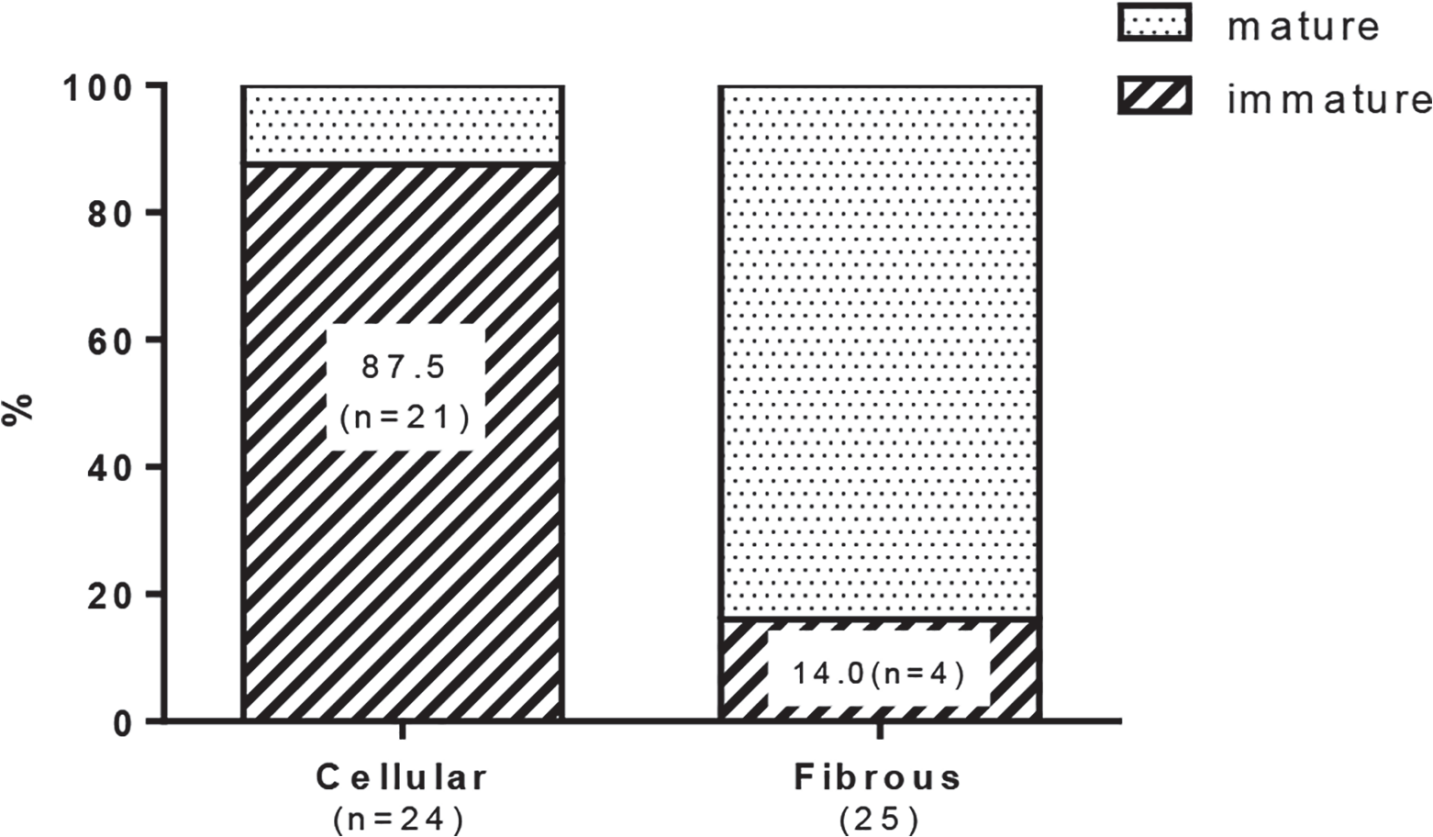
Quantitative analysis of the proportion of mature and immature smooth muscle cell-dominant lesion in the intimal lesions. The lesions in which immature smooth muscle cell is the predominant phenotype of cells (> 50%) accounted for 87.5% (n = 21) in lesions with cellular intimal thickening and 14.0% (n = 4) in lesions with intimal fibrosis (chi-square test, p<.0001). Forty-nine intimal lesions, in which histological classification of the intima and immunophenotyping of smooth muscle cells were performed in serial sections, from 20 rats (n = 4 at 3 week, 4 at 5 week, 6 at 8 week, and 6 at 13 week) were evaluated.

In intimal lesions, we observed 2 phenotypically distinct subtypes of SMCs: hyperchromatic and oval cells staining positive for αSMA, vimentin, SM1 and HHF35 but weakly positive or negative with SM2 and CGA7, representing phenotypically modulated immature SMCs (Fig. 4) and cells staining positive for αSMA, SM1, SM2, HHF35 and CGA7 but weakly positive with vimentin, representing mature SMCs (Fig. 5). Cellular intimal lesions typically comprised immature SMCs, which included CD68-positive intimal macrophages and PCNA-positive cells and were positive for tenascin C (Fig. 4Bi-k). In contrast, intimal fibrosis with dense deposits of elastin comprised mature SMCs, in which few CD68-positive macrophages and PCNA-positive cells were observed (Fig. 5Bij). In the media of small pulmonary arteries, hyperchromatic and oval cells with mature SMC phenotype were occasionally observed (S2 Fig.). Quantitatively, immature SMC-dominant lesions accounted for 87.5% of lesions with cellular intimal thickening and 14.0% of lesions with intimal fibrosis (chi-square analysis, P<.0001) (Fig. 6).

### Myofibroblasts in plexiform lesions

Supporting cells underlying endothelial monolayers in plexiform lesions were αSMA+, vimentin+ myofibroblasts, which were negative for SM1 or SM2 (Fig. 3A, C, D). Typical complex plexiform lesions exhibited remarkable expression of tenascin C, in which numerous CD68-positive macrophages and PCNA-positive cells were observed (Fig. 3Ag-i). In the sprouting plexiform lesion, a complex of an αSMA+ dilatation lesion and supporting cell mass within, each of which was contiguous with αSMA+ medial and intimal SMCs in the parent vessels, was negative for SM1 or SM2 (Fig. 3C, D). Some macrophages infiltrated in the parenchyma surrounding these sprouting lesions (Fig. 3B). By using confocal microscopy, PCNA-positivity was confirmed in αSMA-positive cells in intimal and plexiform lesions; CD68-positive cells did not colocalize with αSMA-positive cells in intimal or plexiform lesions (S3 Fig.).

### Inflammatory cell infiltration and PAH-related gene expression in Sugen/hypoxia rats

**Fig 7:**
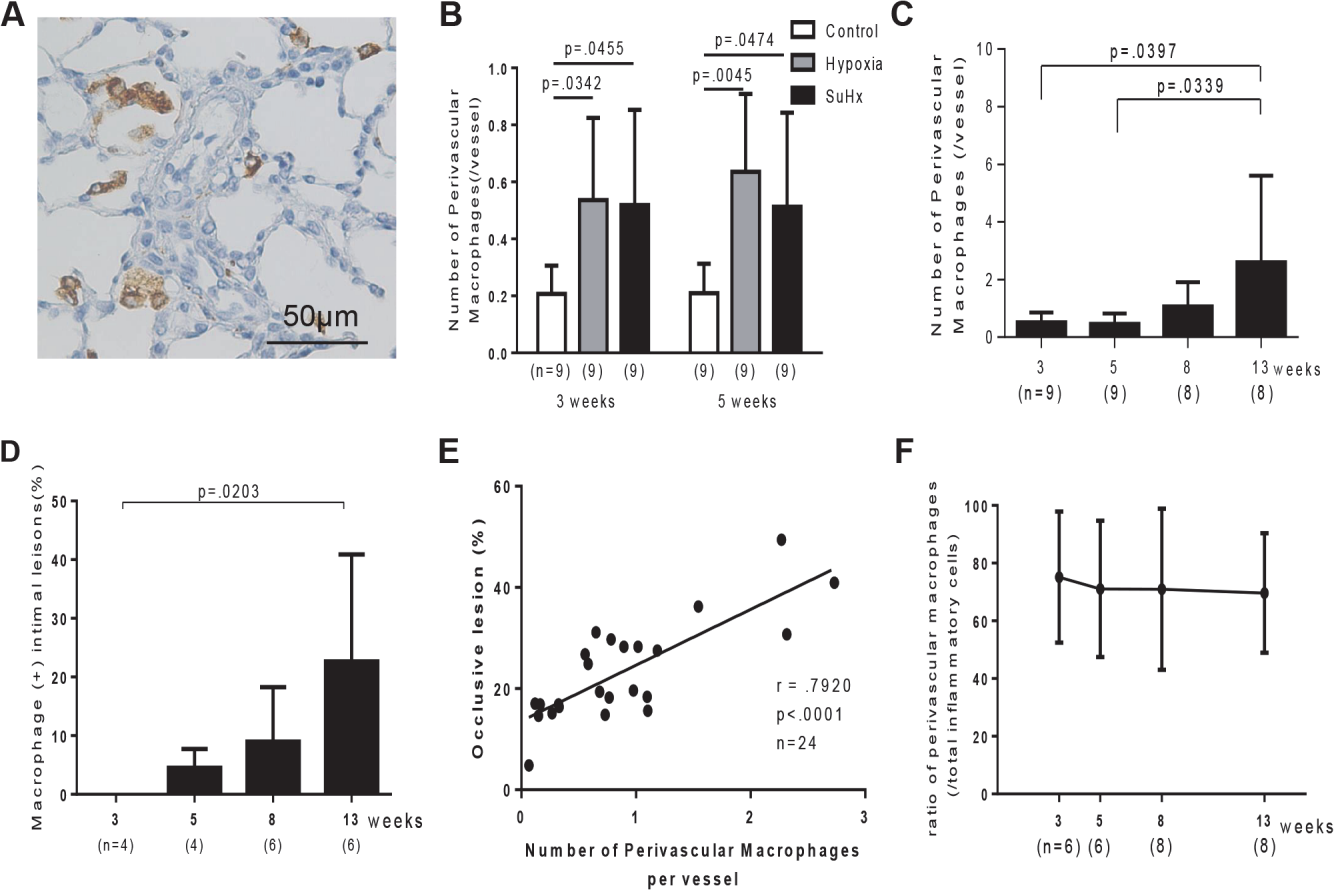
Inflammatory cells in Sugen/hypoxia rats. A: Photomicrographs of CD68-positive macrophages and pulmonary vascular lesions in a Sugen/hypoxia rat 3 weeks after initial treatment. B: Number of perivascular CD68-positive macrophages per vessel in control, hypoxia, and Sugen/hypoxia groups at 3 and 5 weeks was compared with a one-way analysis of variance followed by Tukey-Kramer multiple comparison test. Number of perivascular CD68-positive macrophages per vessel (C) and the percentage of macrophage-positive intima (D) at different time points were compared with a one-way analysis of variance followed by Tukey-Kramer multiple comparison test. Values are mean ± SD. E: Positive correlation between the number of perivascular macrophages per vessel in a lung section and the percentage of occlusive lesions in a lung section (Pearson product-moment correlation coefficients). F: Percentage of the number of perivascular macrophages per vessel in that of total perivascular inflammatory cells, including macrophages, CD3+ T cells, and mast cells, at different time points were compared with a one-way analysis of variance followed by Tukey-Kramer multiple comparison test.

Three and five weeks after initial treatment, perivascular macrophages were significantly increased in Sugen/hypoxia (0.51 ± 0.33, p = .0465 at 3 weeks; 0.51 ± 0.32, p = .0474 at 5 weeks) and in hypoxic rats (0.53 ± 0.28, p = .0342 at 3 weeks; 0.63 ± 0.27, p = .0045 at 5 weeks), compared with in control rats (0.20 ± 0.09 at 3 weeks; 0.20 ± 0.10 at 5 weeks) (Fig. 7A, B). In Sugen/hypoxia rats, the number of perivascular macrophages temporally increased until 13 weeks (2.6 ± 2.9, p = .0397 vs. 3 weeks) (Fig. 7C). Few macrophages infiltrated in the intima at 3 weeks. However, the proportion of macrophage-positive intima temporally increased until 13 weeks (22.6%, p = .0203 vs. 3 weeks) (Fig. 7D). The number of perivascular macrophages was positively correlated with the percentage of occlusive lesions (P<.0001, r = .7920) (Fig. 7E). Perivascular T cells or mast cells were not significantly increased in Sugen/hypoxia and in hypoxic rats at 3 or 5 weeks, compared with in control rats (S4 Fig.). However, in Sugen/hypoxia rats, the number of perivascular T cells (P = .0233 vs. 3 weeks) and mast cells (P<.0001 vs. 3, 5, and 8 weeks) temporally increased until 13 weeks (S4 Fig.). The percentage of the number of perivascular macrophages/ that of total perivascular inflammatory cells, including macrophages, CD3+ T cells, and mast cells, was 75.1% (22.6) at 3 weeks and was constant during the experimental period (Fig. 7F).

**Fig 8:**
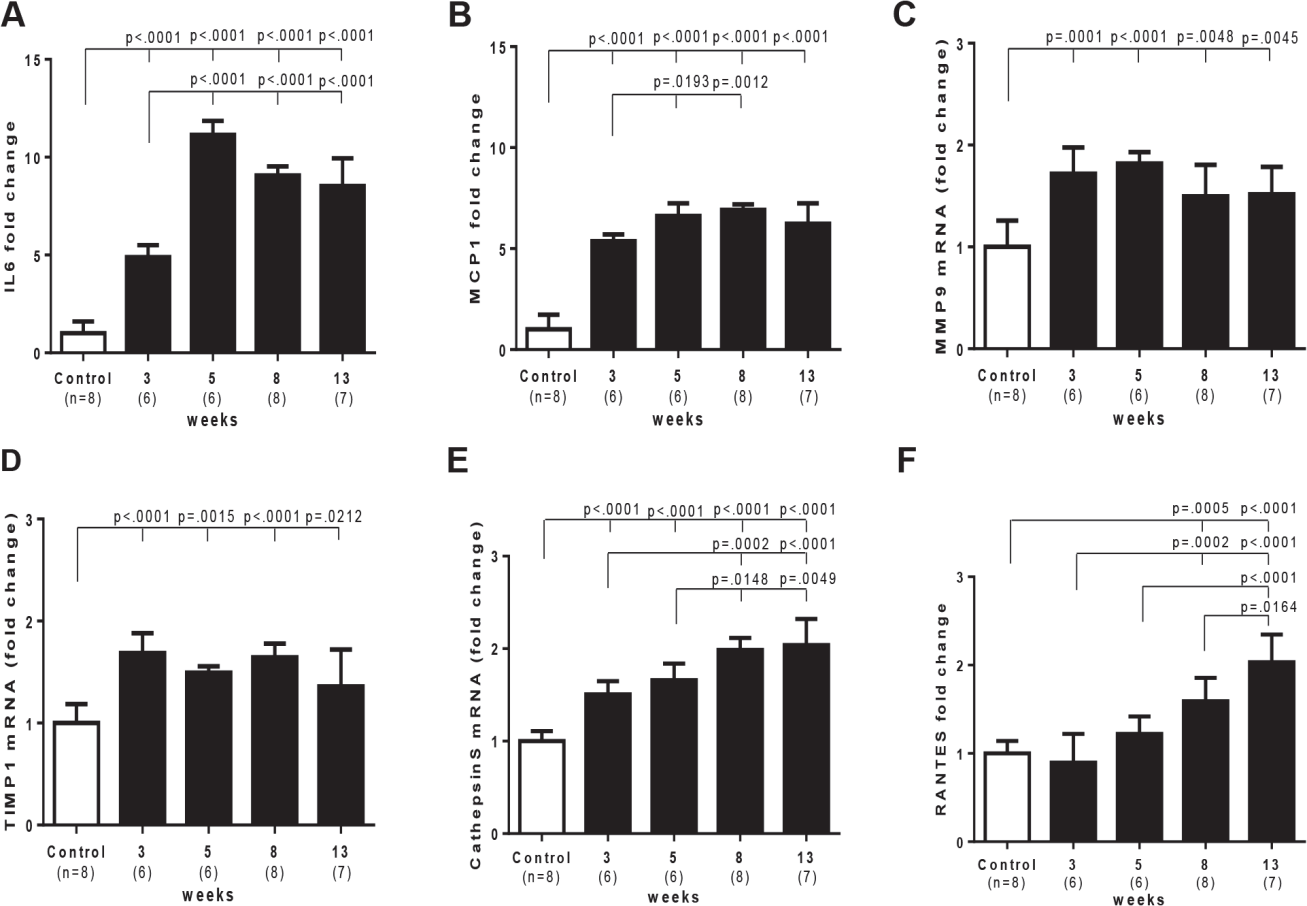
Time course of PAH-related inflammatory gene expression in Sugen/hypoxia rats. Messenger RNA level of various genes at different time points and in controls was compared with a one-way analysis of variance followed by Tukey-Kramer multiple comparison test. Open square (control) indicates the control group 3 weeks after the vehicle treatment; closed square indicates Sugen/hypoxia rats at respective time points. IL6, interleukin 6; MCP1, monocyte chemotactic protein 1; MMP9, matrix metalloproteinase 9; TIMP1, tissue inhibitor of metalloproteinase 1; RANTES, Regulated on Activation, Normal T Cell Expressed and Secreted. Data are expressed as fold-change compared with the control group. Values are mean ± SD.

**Fig 9:**
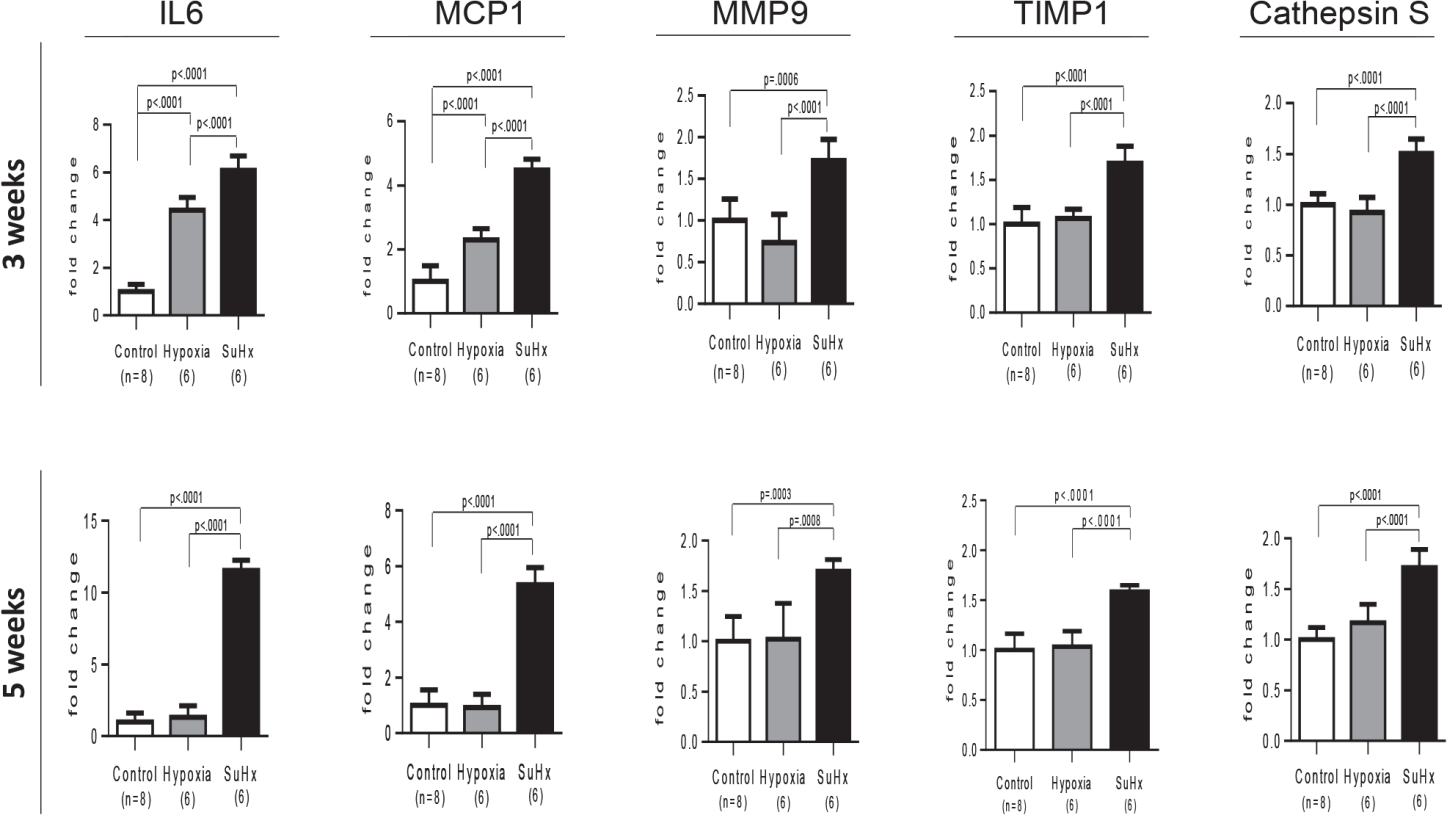
Differential expression of PAH-related inflammatory genes in Sugen/hypoxia rats. Messenger RNA expression level of IL6, MCP1, MMP9, TIMP1, and cathepsin S was compared among Sugen/hypoxia, hypoxia, and control rats at 3 or 5 weeks after initial treatment, with a one-way analysis of variance followed by Tukey-Kramer multiple comparison test. Data are expressed as fold-change compared with the control group. Values are mean ± SD. Abbreviations were described in Fig. 8.

In Sugen/hypoxia rats, mRNA expression of IL6, MCP1, MMP9, TIMP1 and cathepsin S was up-regulated at 3 weeks, which was progressively or persistently up-regulated during the experimental period in the lung, while that of RANTES was increased later at 8 and 13 weeks (Fig. 8). mRNA expression of other molecules, IL-1β, TNFα, VEGF-A, MMP2, and TIMP2 was not consistently up-regulated during the experimental period (S5 Fig.). Compared with in hypoxic or control rats at 3 and 5 weeks, the higher and persistent expression of IL6 and MCP1 and a distinct increase in MMP9 and cathepsin S expression was observed in Sugen/hypoxia rats (Fig. 9). Immunohistochemical and confocal microscopic analyses showed that these inflammatory molecules were expressed in vascular or perivascular inflammatory cells in vascular lesions in this model (S6 Fig.).

## Discussion

**Fig 10:**
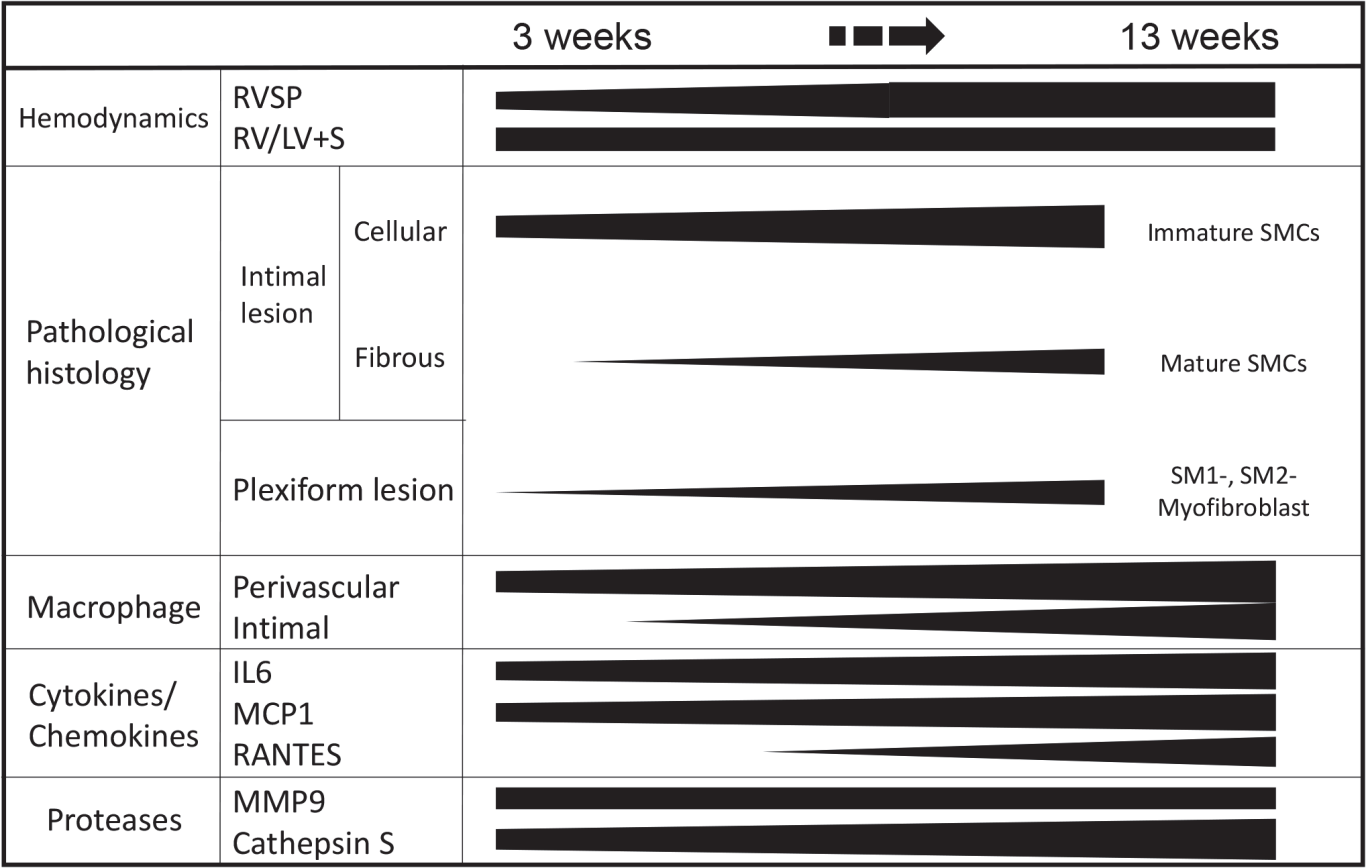
Summary: Phenotypically modulated SMCs and inflammation in the progression of obstructive pulmonary vasculopathy. Abbreviations are described in Figs. 1, 2, and 8.

Amid controversy in the cell type responsible for progressive obstructive pulmonary vasculopathy in human PAH, [1–4] we obtained consistent pathological findings in specific lesions in this model which mimicked previous immunohistochemical findings reported by Yi and Atkinson, [1,2] and electron microscopic findings in human PAH. [3,27,28] We demonstrated that immature and mature SMCs and inflammatory cells, which were previously poorly appreciated in a Sugen/hypoxia rat model, were temporally and topologically associated with the progression of an occlusive pulmonary vasculopathy. We further demonstrated that PAH-related inflammatory genes were progressively or persistently up-regulated and differentially expressed in this progressive model, compared with in the non-progressive model induced by exposure to chronic hypoxia alone. These findings are based on expression pattern of multiple SMC markers, pathological characteristics and gene expression profile in this progressive model. The progression of various pathological parameters and the positive correlation between the percentage of occlusive lesions and RVSP was quantitatively demonstrated in the present study, which is in line with the previous studies of the SuHx model and human PAH [16,29,30]. An early increase (at 3–8 weeks) in RVSP and the RV/LV+S ratio in spite of the later peak (at 13 weeks) in the indices of occlusive vasculopathy could be related to the vasoconstrictive nature of vessels in the early stage of disease (at 3–5 weeks) and the later decrease in cardiac output, which is associated with advanced pulmonary vasculopathy, as in previous studies [29,31], suggesting uncertainty in the causality between an increase in RVSP and the development of occlusive vasculopathy. The present findings are summarized in a schematic diagram (Fig. 10).

### Immunohistochemistry for phenotyping SMCs in the intima

Intimal lesions comprised supporting cell mass that was lined by endothelial monolayers. These findings are consistent with a recent report on the Sugen/hypoxia model, [16] although such supporting cells were poorly characterized, [16] and the role of apoptosis-resistant endothelial cells has been appreciated in a series of previous studies. [19,20] We found that the intimal lesions comprised immature and mature SMCs. Although many SMC markers, including αSMA, are not regarded as SMC-specific markers and there is no single marker currently available for rigorously identifying phenotypically modulated SMC, recent experimental and clinical studies have shown that smooth muscle-myosin heavy chain isoforms are specifically expressed by SMC: an alternatively spliced variant of the smooth muscle-myosin heavy chain (SM2) is expressed mainly by differentiated SMCs, while another smooth muscle heavy chain marker SM1 represents both differentiated and dedifferentiated SMC. [5,6,8,9,11,32] Thus, the present immunohistochemical analysis, using multiple SMC markers SM1 and SM2, as well as SMC actin markers HHF35 and CGA7, in series of specimens, consistently showed phenotypically modulated immature and mature SMCs in the intimal lesions, as reported for other vascular diseases. [8,9,10,14,15] These findings are consistent with electron microscopy-based SMC-like cells or αSMA+, vimentin+ myofibroblasts in intimal lesions in PAH patients and SM1+, SM2+/- immature SMCs in the intimal lesions in a case report of PAH. [1,2,15,28,33]

Conversely, recent evidence demonstrated that endothelial cells can transition into mesenchymal cells expressing αSMA or smooth muscle-myosin heavy chain markers, that intimal lesions in pulmonary hypertension contained some cells co-expressing VWF and αSMA in vivo, and that this process can contribute to the accumulation of SMCs in pulmonary vascular diseases. [34,35] However, we did not observe such double positive cells in intimal lesions in the confocal immunofluorescent studies. In addition, it is also reported that pericytes, adventitial fibroblasts, or circulating or local progenitors could differentiate into αSMA+ cells in pulmonary vasculopathy. [36,37] Although hyperchromatic and oval cells with mature SMC phenotype observed in the media of small vessels could be a potential source, the present study can never exclude the possibility that the ‘origin’ of the immature SMCs is other cell types, including endothelial cells, pericytes, perivascular fibroblasts, and circulating or local progenitors.

### Immature and mature SMCs in the progression of intimal lesions

Consistent with a progressive increase in indices of cellular intimal thickening, immature SMCs in cellular intimal lesions exhibited a proliferating (represented by PCNA positivity), pro-inflammatory (represented by infiltration of macrophages) and secretory phenotype (represented by matrix remodeling and fragmentation of elastic laminae). Such findings were supported by the expression of MCP1, IL6, MMP9, cathepsin S and tenascin C in lungs in the present study, and other reports on human PAH. [38,39] Therefore, immature SMCs may contribute to the progression of obliterating vasculopathy observed in this model, as shown for other fibroproliferative diseases. [8,9,10,14] In an advanced fibrous intimal lesion, supporting intimal cells primarily exhibited a mature phenotype of SMC. For the intimal lesion after experimental balloon injury, SM1-, SM2- very immature myofibroblasts preceded the appearance of SM1+, SM2- phenotypically modulated SMCs, which were followed by SM1+, SM2+ mature SMCs. [8] Therefore, dedifferentiated SMCs located in cellular intimal lesions may redifferentiate into the mature phenotype in fibrous intima in the present model, as in electron microscopic findings of human PAH and in our case report of PAH. [15,33]

### SM1-, SM2- myofibroblasts in plexiform lesions

In the present study, we observed that the plexiform lesion comprised a complex plexus of aneurysmal and supporting cell mass with vascular channels that were lined by monolayers of endothelial cells, although vascular channels were sparsely distributed and were separated by abundant αSMA+ supporting cell cluster and matrix deposition in some complex plexiform lesions. These findings were consistent with previous studies using this model as well as in human PAH, [1,3,16,28] although supporting cells were poorly characterized in this model. We found that aneurysmal and supporting cells in such complex lesions represent the features of very immature αSMA+, vimentin+ myofibroblasts that were negative for SM1 or SM2, with proliferative, pro-inflammatory and secretory properties. These findings were consistent with human PAH in previous reports. [1–3,38] In sprouting lesions, we found that αSMA+, SM1-, SM2- supporting cell mass and the dilatation lesion, which was associated with diminished elastic laminae, appeared to be contiguous with αSMA+ intimal and medial SMCs in the parent vessel, respectively. It is therefore possible that supporting cells in the intimal and sprouting plexiform lesions may be pathologically from a similar origin, as shown in human PAH, and that phenotypic modulation of SMC in this model may be related to the fragility of the vascular wall in aneurysmal lesions. [1,3,7,28]

### Macrophage infiltration and PAH-related inflammatory gene expression

In the Sugen/hypoxia model, we found macrophages infiltrated in the perivascular space and intimal lesions, which have been poorly characterized in a series of previous studies. [16,19,20,29] Although the positive correlation between the number of perivascular macrophages and an increase in the intima-media complex in human PAH was recently reported, [30] the temporal relationship between obstructive pulmonary vasculopathy and inflammatory cell infiltration was obviously unknown in human. We found the temporal increase in perivascular and intimal macrophages and occlusive vasculopathy, as well as the positive correlation between the proportion of occlusive lesions and the number of perivascular macrophages, suggesting that the progressive obstructive vasculopathy in this model was temporally associated with an increase in inflammatory cells in the lesions. However, there was no difference in the number of perivascular macrophages between hypoxia alone vs. Sugen/Hypoxia rats at 3 and 5 weeks, suggesting that macrophage accumulation alone cannot explain the difference in pulmonary vascular remodeling between them. Although the number of other inflammatory cells, CD3+ T cells and mast cells, also significantly increased until 13 weeks in this model, there were no significant increase in the number of these cells in Sugen/hypoxia rats or hypoxia rats at 3 and 5 weeks, compared with that in controls, suggesting accumulation of CD3+ T cells or mast cells alone cannot explain the difference between Sugen/hypoxia and hypoxia rats either. Therefore, potential difference in gene expression profile or any cellular function in macrophages or other inflammatory cells between Sugen/hypoxia rats and hypoxia only rats may be relevant to the occlusive remodeling process in diseases.

Next, we investigated whether such progressive vasculopathy is temporally associated with expression of inflammatory gene in the present model, which were previously correlated to the pathogenesis of PAH and/or the elastolytic change of vessels. [23,30,38] We found that the progression of pulmonary vasculopathy was in parallel with early and progressive or persistent upregulation of IL6, MCP1, cathepsin S, and MMP9 and later upregulation of RANTES. [7,38,40] Furthermore, we investigated whether such PAH-related inflammatory genes are differentially expressed in this model in comparison with a ‘non-progressive’ model. We found that this progressive model was in fact characterized by the higher and persistent expression of inflammatory molecules (IL6, MCP1, MMP9 and cathepsin S) in the lungs, each of which was localized in obstructive vascular lesions in this model. These findings suggest that the progressive nature of pulmonary vasculopathy in this model may be related to such inflammatory mechanisms, which could function upstream and/or downstream of phenotypically modulated SMCs in the Sugen/hypoxia model. [5,6,12,13,39,41–43]

Amid controversy in cellular components for each lesions in human PAH, this is the first study demonstrating that immature SMCs and related inflammation are associated with the progressive nature of obstructive pulmonary vasculopathy in Sugen/hypoxia rats. The present observational study using the Sugen/hypoxia model may give an insight into the cellular basis of intractable lesions in human PAH.

## Supporting Information

S1 Table Immunophenotyping of smooth muscle cells. (PDF)

S2 Table Number of animals for each group in Fig. 1A. (PDF)

S1 Fig Remnants of elastic laminae and vascular channels in complex plexiform lesion. Photomicrographs of low magnification (Panel A, a marked panel for Fig. 3Aa) and high magnification (Panel B) of a complex plexiform lesion in a rat 13 weeks after initial treatment in EVG staining. Photomicrographs of low magnification (Panel C, a marked panel for Fig. 3Ab) and high magnification (Panel D) of the same complex plexiform lesion in immunohistochemical analysis for von Willebrand factor. An arrow head indicates fragments of elastic laminae; an arrow, von Willebrand factor-positive endothelial cell monolayers. Abbreviations are described in Fig. 2.
(TIF)

S2 Fig Mature smooth muscle cells in the media of small pulmonary arteris in Sugen/hypoxia rats. Photomicrographs of cross-sectional sections in the media of small pulmonary arteries, including hyperchromatic and oval cells, in Sugen/hypoxia rats. Immunohistochemical findings using antibodies for various antibodies were presented. Hyperchromatic and oval cells staining positive for αSMA, SM1, SM2, HHF35 and CGA7 were regarded as representing mature smooth muscle cells. Abbreviations were described in Fig. 2.
(TIF)

S3 Fig Immunolocalization of αSMA-positive cells and PCNA-positive cells or CD68-positive macropahges in intimal and plexiform lesions. Photomicrographs of a sprouting intimal lesion (Panels Aa-d), an intimal lesion (Panels Ae-h and Ba-d), and a plexiform lesion (Panels Ai-l and Be-h). Immunolocalization of αSMA-positive cells and PCNA-positive cells or CD68-positive macropahges in intimal and plexiform lesions, as evaluated by confocal microscopy, is shown. Abbreviations were described in Figs. 2 and 3.
(TIF)

S4 Fig Other inflammatory cells in Sugen/hypoxia rats. Panel A: Photomicrographs of perivascular CD3-positive T cells in a Sugen/hypoxia rat. Panel B: Number of perivascular T cells per vessel in control, hypoxia, and Sugen/hypoxia groups at 3 or 5 weeks was compared with a one-way analysis of variance followed by Tukey-Kramer multiple comparison test. Panel C: Number of perivascular T cells per vessel at different time points was compared with a one-way analysis of variance followed by Tukey-Kramer multiple comparison test. Panel D: Photomicrographs of perivascular toluidine blue-positive mast cells in a Sugen/hypoxia rat. Panel E: Number of perivascular mast cells per vessel in control, hypoxia, and Sugen/hypoxia groups at 3 or 5 weeks was compared with a one-way analysis of variance followed by Tukey-Kramer multiple comparison test. Panel F: Number of perivascular mast cells per vessel at different time points was compared with a one-way analysis of variance followed by Tukey-Kramer multiple comparison test.
(TIF)

S5 Fig Time course of other inflammatory gene expression. Messenger RNA expression level of interleukin 1β (IL1β), tumor necrosis factor α (TNFα), vascular endothelial growth factor A (VEGF A), matrix metalloproteinase 2 (MMP2), and tissue inhibitor of metalloproteinase 2 (TIMP2) was compared at different time points and controls, with a one-way analysis of variance followed by Tukey-Kramer multiple comparison test. Open square (control) indicates the control group 3 weeks after the vehicle treatment; closed square indicates Sugen/hypoxia rats at the respective time point. Data are expressed as fold-change compared with the control group. Values are mean ± SD.
(TIF)

S6 Fig Expression of PAH-related inflammatory and proteolytic molecules in pulmonary vascular lesions in Sugen/hypoxia rats. Photomicrographs of immunohistochemical (Panels A), and immunofluorescent confocal microscopic findings (Panels B) of vessels with intimal lesions in SuHx rats using various antibodies were shown. Photomicrographs of immunofluorescent confocal microscopic findings, using antibodies for MCP1 and αSMA, were presented in control rats (Panels Ca-d), in hypoxia rats (Panels Ce-l), and in SuHx rats (Panels Cm-t) at 3 and 5 weeks. IL6 and MCP1 were expressed in intima and hypertrophied media, and cathepsin S and RANTES were expressed in perivascular inflammatory cells (Panel A); MMP9 was expressed in αSMA-negative cells in hypertrophied media and intima (Panel B). MCP1 was mainly expressed in αSMA-positive cells in intimal and plexiform lesion, as well as hypertrophied media in Sugen/hypoxia rats, less and transiently expressed in hypertrophied media in hypoxic rats, and very weakly expressed in media in controls (Panel C). Abbreviations were described in Figs. 1, 2 and 8.
(TIF)
