# Supplementary material for: Potential Contribution of Phenotypically Modulated Smooth Muscle Cells and Related Inflammation in the Development of Experimental Obstructive Pulmonary Vasculopathy in Rats
Source: PLoS One. 2015 Feb 25;10(2):e0118655. doi: 10.1371/journal.pone.0118655 (PMC4340876; doi:10.1371/journal.pone.0118655)
Supplement: S1 Table — (PDF) [file pone.0118655.s001.pdf]

Table S1. Immunophenotyping of smooth muscle cells

	MHC markers		Actin markers		
SMC phenotype	SM1	SM2	αSMA	HHF35	CGA7
Mature	+	+	+	+	+
Immature	+	+/-	+	+	+/-

Definition of abbreviations: MHC = myosin heavy chain. SMC = smooth muscle cell. αSMA = α smooth muscle actin.
