# Supplementary material for: Potential Contribution of Phenotypically Modulated Smooth Muscle Cells and Related Inflammation in the Development of Experimental Obstructive Pulmonary Vasculopathy in Rats
Source: PLoS One. 2015 Feb 25;10(2):e0118655. doi: 10.1371/journal.pone.0118655 (PMC4340876; doi:10.1371/journal.pone.0118655)
Supplement: S2 Table — (PDF) [file pone.0118655.s002.pdf]

Table S2. Number of animals used for each group in Figure 1A

	Weeks after treatment*	n**
SuHx	0-3	28
	4-5	22
	6-8	16
	9-13	8
Hypoxia	0-3	10
	4-5	5
Control	0-3	10
	4-5	5

Definition of abbreviations: SuHx = Sugen/hypoxia.

* Weeks after treatment: Sugen or diluent treatment.

**n: number of rats.
