# Supplementary figures and images for: Potential Contribution of Phenotypically Modulated Smooth Muscle Cells and Related Inflammation in the Development of Experimental Obstructive Pulmonary Vasculopathy in Rats
Source: PLoS One. 2015 Feb 25;10(2):e0118655. doi: 10.1371/journal.pone.0118655 (PMC4340876; doi:10.1371/journal.pone.0118655)

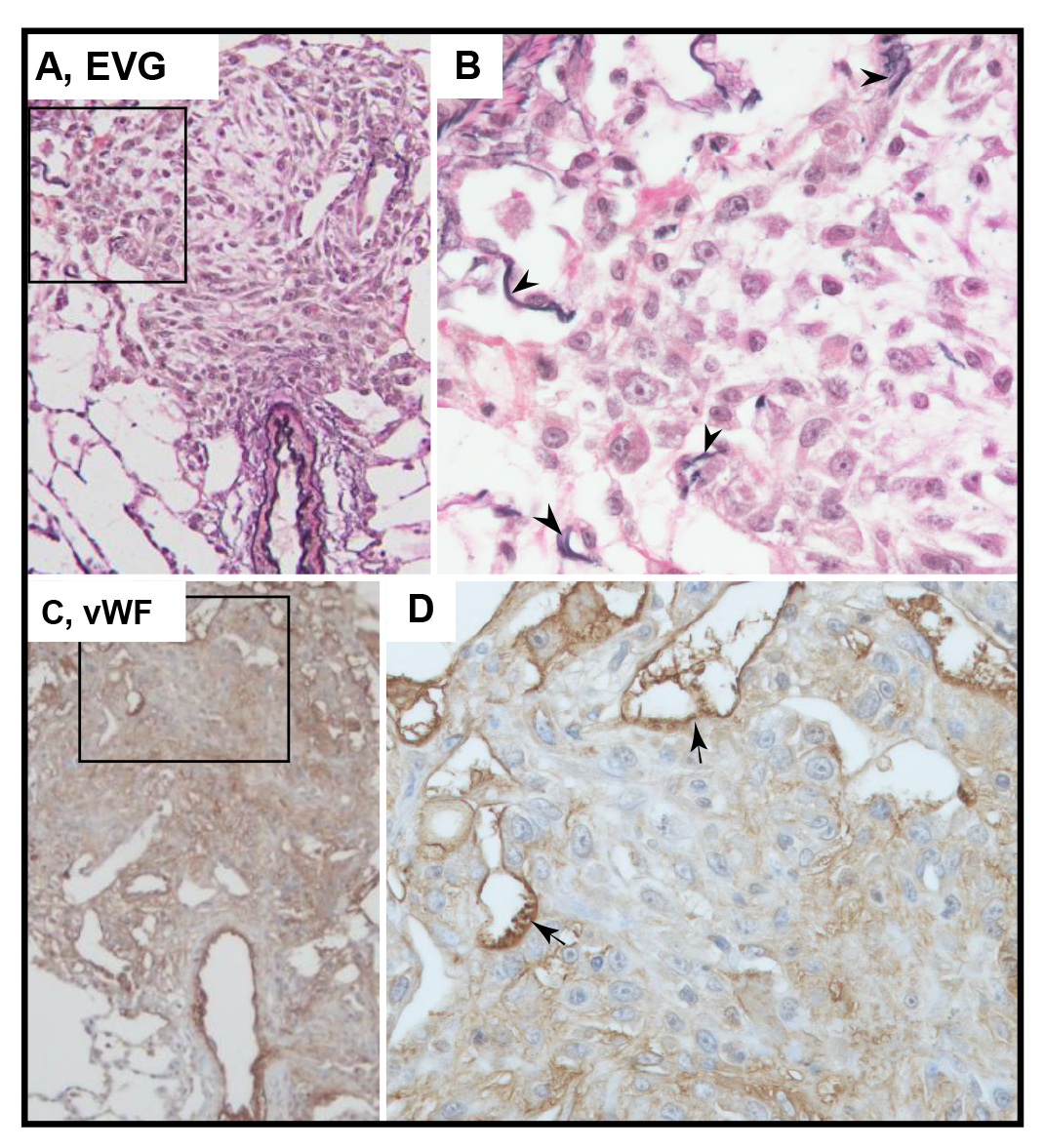

Supplement: S1 Fig — Photomicrographs of low magnification (Panel A, a marked panel for Fig. 3Aa) and high magnification (Panel B) of a complex plexiform lesion in a rat 13 weeks after initial treatment in EVG staining. Photomicrographs of low magnification (Panel C, a marked panel for Fig. 3Ab) and high magnification (Panel D) of the same complex plexiform lesion in immunohistochemical analysis for von Willebrand factor. An arrow head indicates fragments of elastic laminae; an arrow, von Willebrand factor-positive endothelial cell monolayers. Abbreviations are described in Fig. 2. (TIF) [file pone.0118655.s003.tif]

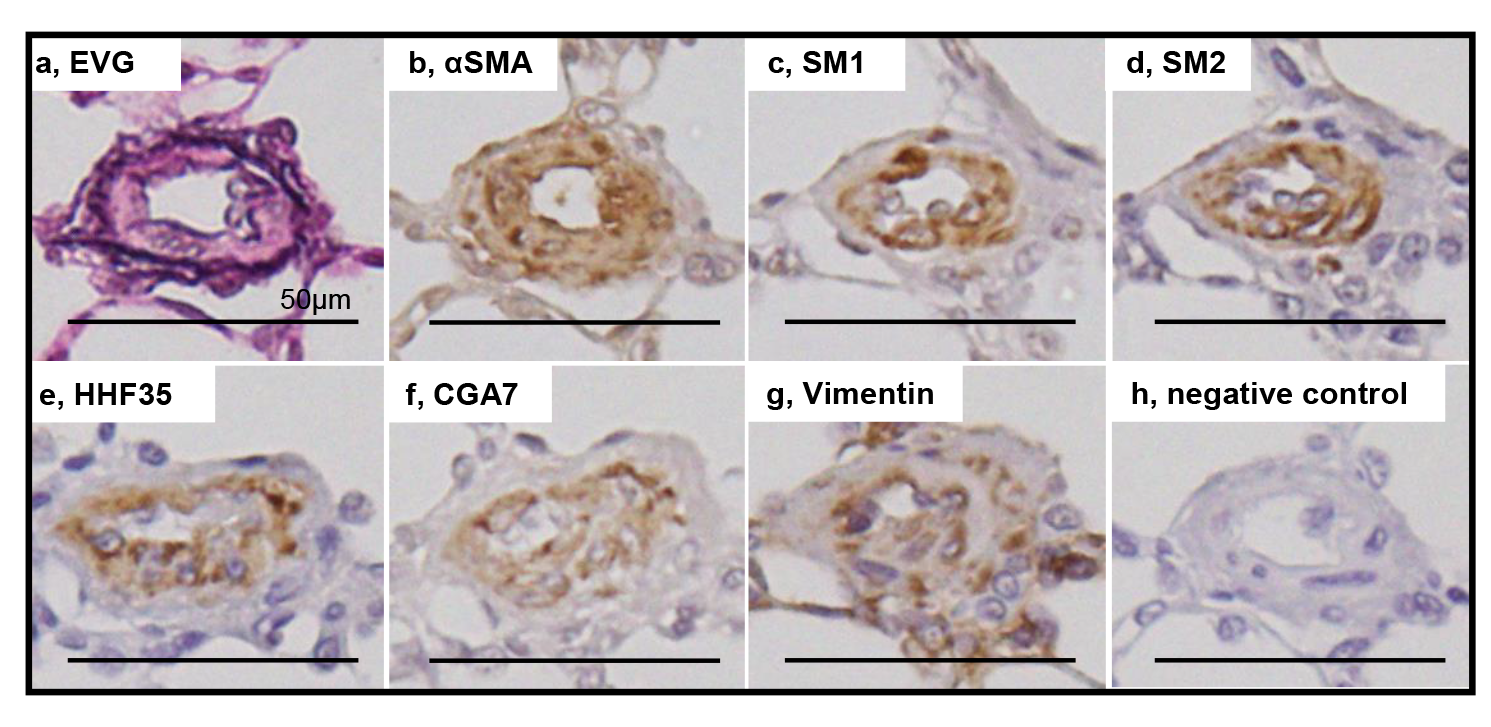

Supplement: S2 Fig — Photomicrographs of cross-sectional sections in the media of small pulmonary arteries, including hyperchromatic and oval cells, in Sugen/hypoxia rats. Immunohistochemical findings using antibodies for various antibodies were presented. Hyperchromatic and oval cells staining positive for αSMA, SM1, SM2, HHF35 and CGA7 were regarded as representing mature smooth muscle cells. Abbreviations were described in Fig. 2. (TIF) [file pone.0118655.s004.tif]

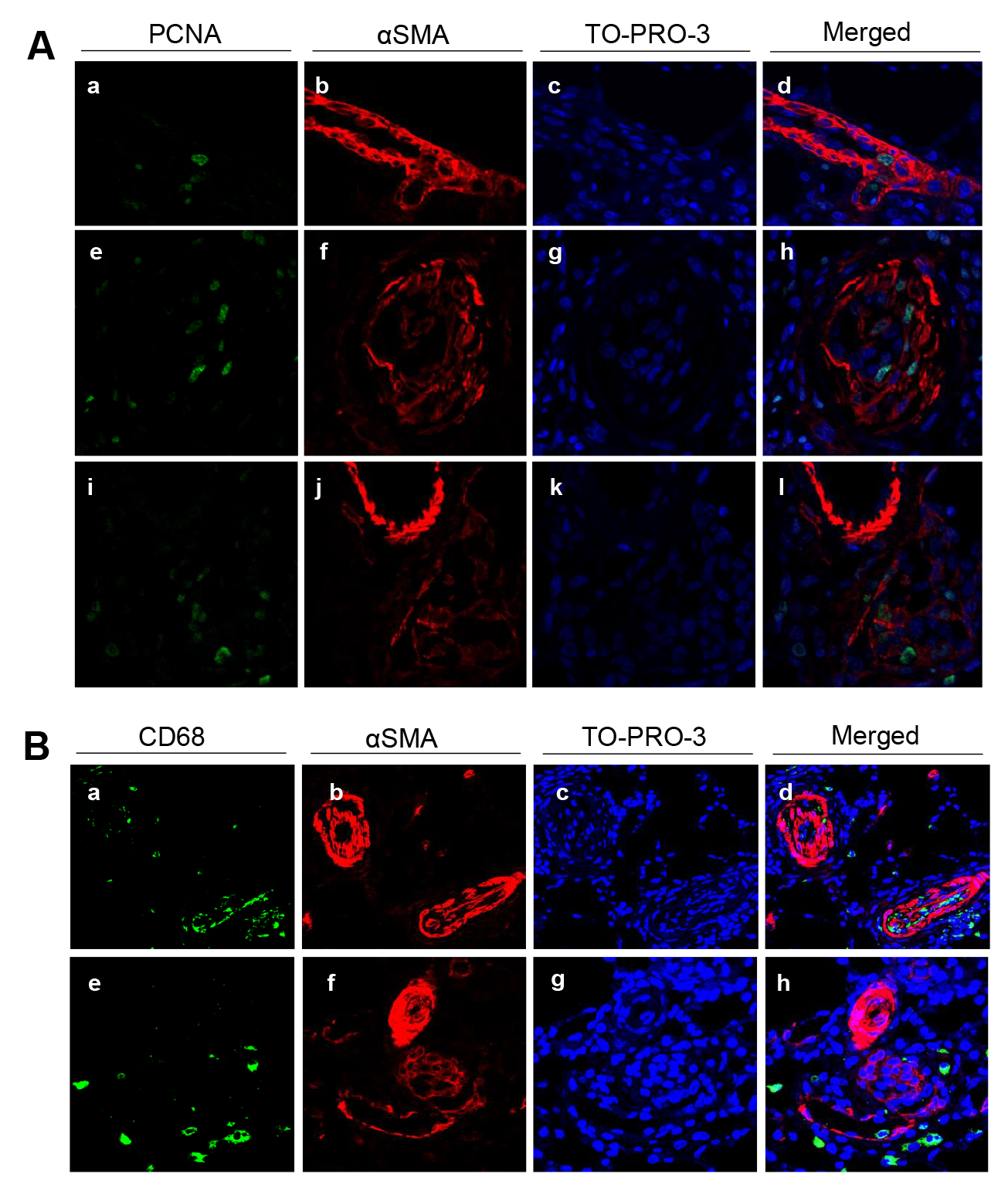

Supplement: S3 Fig — Photomicrographs of a sprouting intimal lesion (Panels Aa-d), an intimal lesion (Panels Ae-h and Ba-d), and a plexiform lesion (Panels Ai-l and Be-h). Immunolocalization of αSMA-positive cells and PCNA-positive cells or CD68-positive macropahges in intimal and plexiform lesions, as evaluated by confocal microscopy, is shown. Abbreviations were described in Figs. 2 and 3. (TIF) [file pone.0118655.s005.tif]

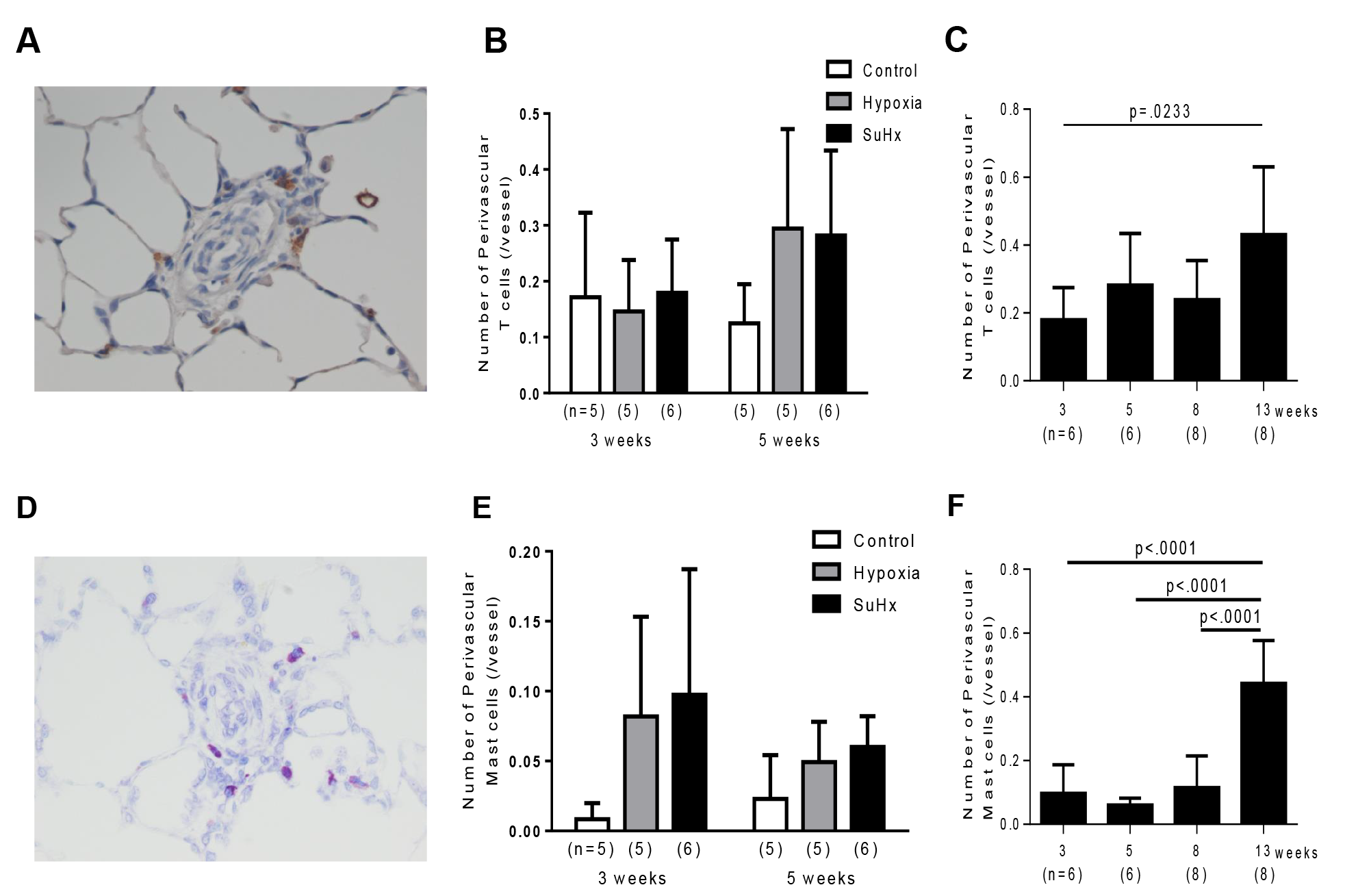

Supplement: S4 Fig — Panel A: Photomicrographs of perivascular CD3-positive T cells in a Sugen/hypoxia rat. Panel B: Number of perivascular T cells per vessel in control, hypoxia, and Sugen/hypoxia groups at 3 or 5 weeks was compared with a one-way analysis of variance followed by Tukey-Kramer multiple comparison test. Panel C: Number of perivascular T cells per vessel at different time points was compared with a one-way analysis of variance followed by Tukey-Kramer multiple comparison test. Panel D: Photomicrographs of perivascular toluidine blue-positive mast cells in a Sugen/hypoxia rat. Panel E: Number of perivascular mast cells per vessel in control, hypoxia, and Sugen/hypoxia groups at 3 or 5 weeks was compared with a one-way analysis of variance followed by Tukey-Kramer multiple comparison test. Panel F: Number of perivascular mast cells per vessel at different time points was compared with a one-way analysis of variance followed by Tukey-Kramer multiple comparison test. (TIF) [file pone.0118655.s006.tif]

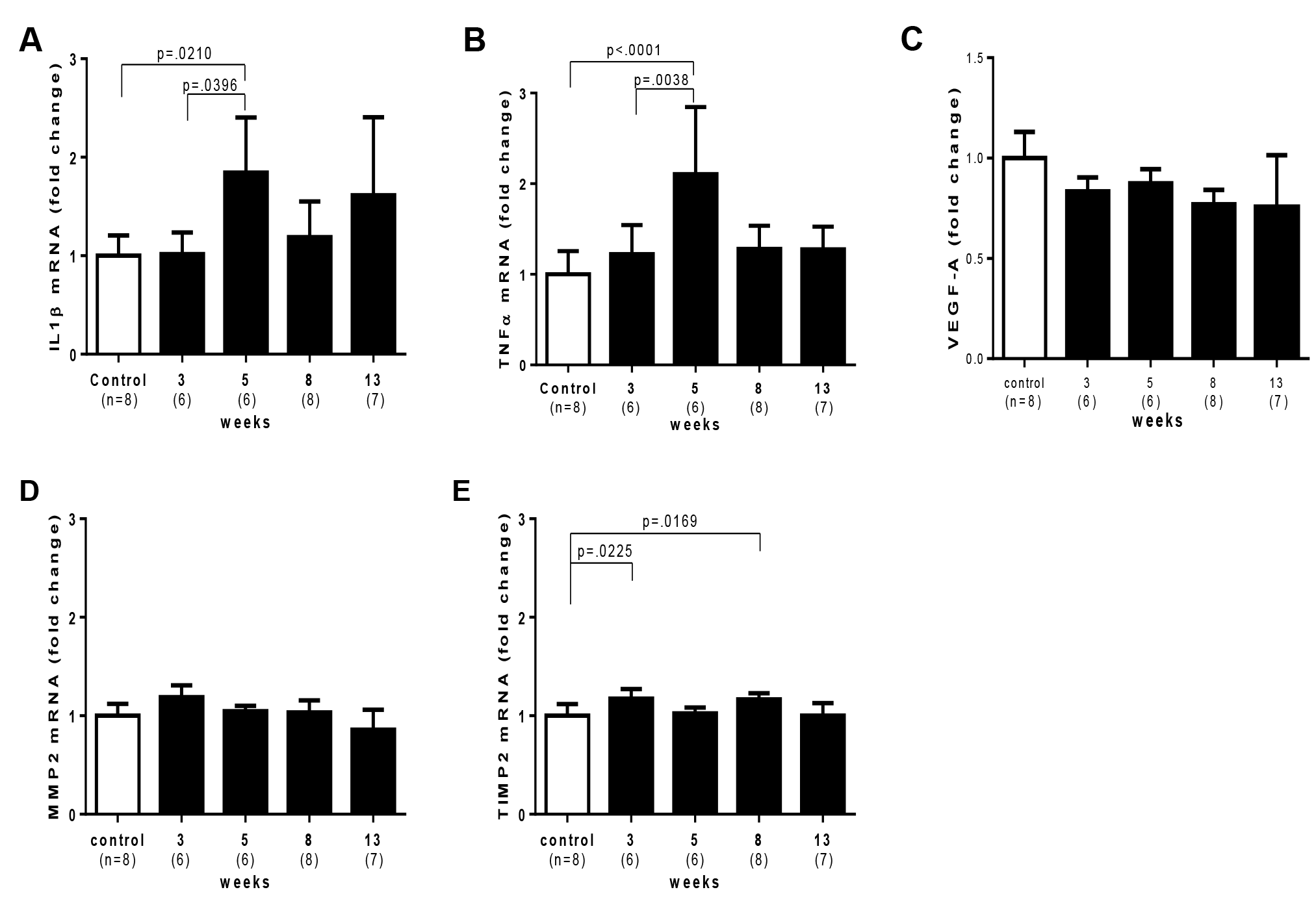

Supplement: S5 Fig — Messenger RNA expression level of interleukin 1β (IL1β), tumor necrosis factor α (TNFα), vascular endothelial growth factor A (VEGF A), matrix metalloproteinase 2 (MMP2), and tissue inhibitor of metalloproteinase 2 (TIMP2) was compared at different time points and controls, with a one-way analysis of variance followed by Tukey-Kramer multiple comparison test. Open square (control) indicates the control group 3 weeks after the vehicle treatment; closed square indicates Sugen/hypoxia rats at the respective time point. Data are expressed as fold-change compared with the control group. Values are mean ± SD. (TIF) [file pone.0118655.s007.tif]

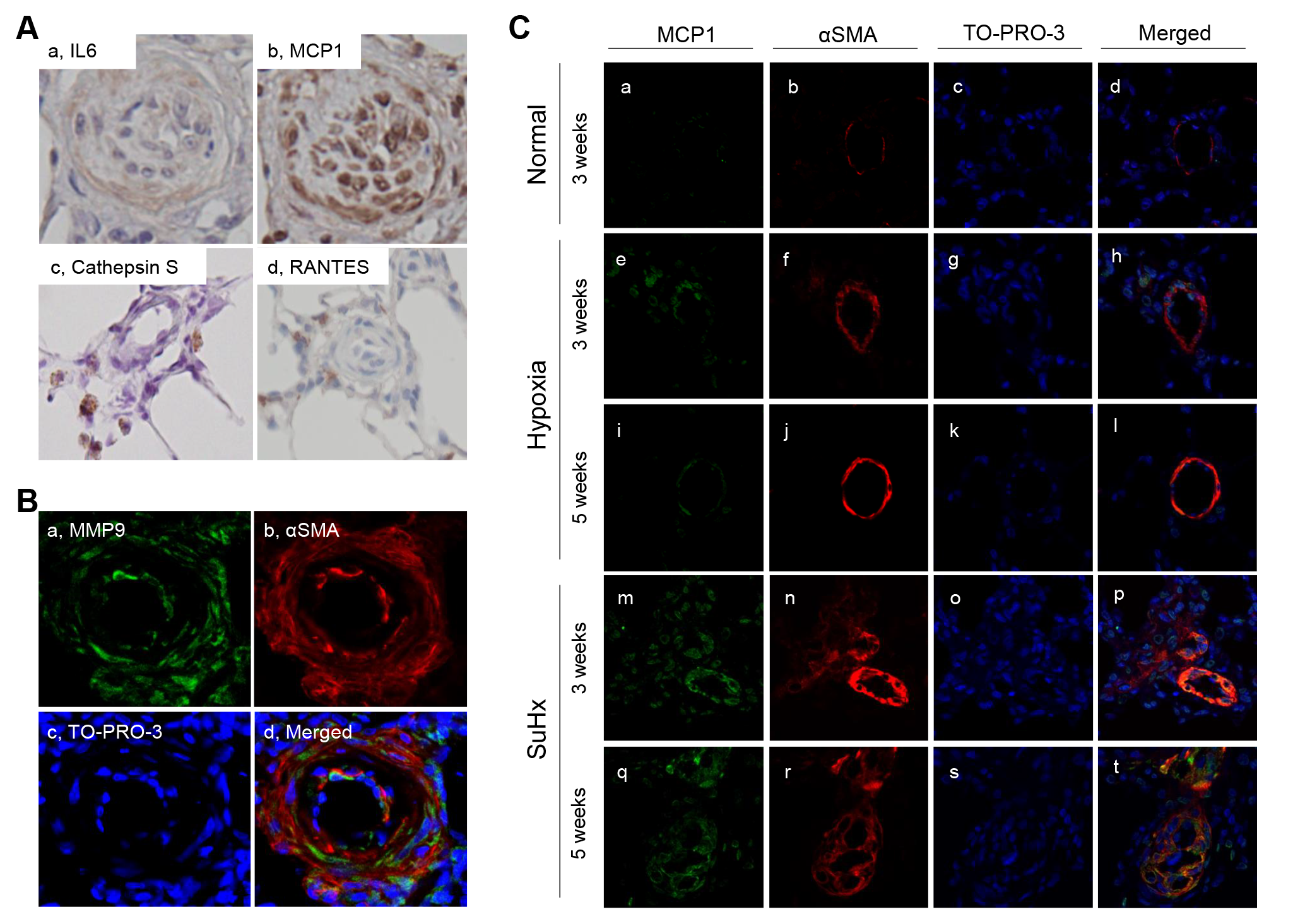

Supplement: S6 Fig — Photomicrographs of immunohistochemical (Panels A), and immunofluorescent confocal microscopic findings (Panels B) of vessels with intimal lesions in SuHx rats using various antibodies were shown. Photomicrographs of immunofluorescent confocal microscopic findings, using antibodies for MCP1 and αSMA, were presented in control rats (Panels Ca-d), in hypoxia rats (Panels Ce-l), and in SuHx rats (Panels Cm-t) at 3 and 5 weeks. IL6 and MCP1 were expressed in intima and hypertrophied media, and cathepsin S and RANTES were expressed in perivascular inflammatory cells (Panel A); MMP9 was expressed in αSMA-negative cells in hypertrophied media and intima (Panel B). MCP1 was mainly expressed in αSMA-positive cells in intimal and plexiform lesion, as well as hypertrophied media in Sugen/hypoxia rats, less and transiently expressed in hypertrophied media in hypoxic rats, and very weakly expressed in media in controls (Panel C). Abbreviations were described in Figs. 1, 2 and 8. (TIF) [file pone.0118655.s008.tif]
